# Simulation of bronchial airway acoustics in healthy and asthmatic subjects

**DOI:** 10.1371/journal.pone.0228603

**Published:** 2020-02-10

**Authors:** Lorenzo Aliboni, Francesca Pennati, Thomas J. Royston, Jason C. Woods, Andrea Aliverti

**Affiliations:** 1 Dipartimento di Elettronica, Informazione e Bioingegneria, Politecnico di Milano, Milan, Italy; 2 Richard and Loan Hill Department of Bioengineering, University of Illinois at Chicago, Chicago, Illinois, United States of America; 3 Department of Pediatrics, Department of Radiology, University of Cincinnati, Cincinnati, Ohio, United States of America; 4 Department of Physics, Washington University, St Louis, Missouri, United States of America; University of Genova, ITALY

## Abstract

The onset and development of many airway pathologies affect sound propagation throughout the respiratory system; changes in respiratory sounds are detected primarily by auscultation, which is highly skill dependent. The aim of the present study was to compare healthy and asthmatic pulmonary acoustics by applying a 1D model of wave propagation on CT-based patient-specific geometries. High-resolution CT lung images were acquired in five healthy volunteers and five asthmatic patients at total lung capacity (TLC) and functional residual capacity (FRC). Tracheobronchial trees were reconstructed from CT images. Acoustic pressure, impedance and wall radial velocity were measured by simulating acoustic wave propagation of two external, acoustic pressure waves (1 Pa, 200 and 600 Hz) from the trachea level to the 4th generation. In asthmatic patients, acoustic pressure averaged across the last three generations showed a reduction equal to 29.7% (p<0.01) at FRC, at 200 Hz; input and terminal impedance were 34.5% (p<0.05) higher both at FRC and TLC; wall radial velocity was more than 80% (p<0.05) lower in higher generations both at FRC and TLC. Airway differences in asthma alter acoustic parameters at FRC and TLC, with the greatest difference at FRC and 200 Hz. Acoustic wave propagation analysis represents a quantitative approach that has potential to objectively characterize airway differences in individuals with diseases such as asthma.

## Introduction

Auscultation has been used by clinicians for hundreds of years to qualitatively diagnose and monitor the progression of pulmonary pathologies. Auscultation technique is based on the clinical evidence that the morphological and functional alterations of the respiratory system result in measurable changes in sound generation and propagation [1]. Over the last few decades an increasing interest in the quantitative and objective characterization of the sound generation and propagation in the respiratory system has led to a concerted effort towards the development of proper mechano-acoustical models to represent these phenomena.

Wodicka et al. [2] described an acoustic model of the respiratory system based on an analogy with a model of electrical distributed parameters to evaluate sound propagation from inside the respiratory tract to the thoracic surface. Suki et al.[3] and Jackson et al. [4,5] studied the frequency dependency of acoustic impedance of the subglottal respiratory tract. Habib et al. [6] identified the constituent relations to relate the alterations of the acoustic impedance to the airway geometry and the airway wall mechanical properties. In Royston et al. [7] sound propagation from trachea to thoracic surface in healthy and pneumothorax conditions was simulated. Dai et al. [8] and Yeng et al. [9,10]studied both numerically and experimentally the sound transmission in the lungs. Lulich et al. [11,12] focused on the analysis of resonance phenomena and wave propagation velocities in the lower respiratory tract. Henry et al. [13] further extended the approach in [8,14] to account for image-based geometries in healthy subjects and proposed a simplified approach to specific pathological conditions. In the present work, we applied the analytical model developed by Henry et al. [13] to study the sound propagation in real tracheobronchial trees reconstructed from CT data in healthy and asthmatic subjects.

In the previous approach asthma pathology was simulated using the hypothesis proposed in Ionescu at al. [15]; the approach presented in this study is based on patient-specific geometries that account for the complexity and heterogeneity of the pathology [16,17].

The objective of the present study is to demonstrate that the morphological alterations present in asthma determine a detectable and quantifiable variation of the acoustic parameters of the tracheobronchial tree. More specifically, we aim to 1) apply the acoustic model to tracheobronchial trees reconstructed from CT data in health and asthma; 2) compare the response of the system to an inlet acoustic pressure of known amplitude in terms of acoustic pressure distribution, acoustic impedance and acoustic wall radial velocity over different frequencies.

## Materials and methods

### Study subjects

Five severe asthmatic subjects and five healthy volunteers (no history of pulmonary disease or abnormalities) underwent CT imaging in the supine position and the data were acquired during breath-hold at functional residual capacity (FRC) and at total lung capacity (TLC). Anthropometric and spirometry data for the two study groups are reported in Table 1.

**Table 1:** Baseline demographics and data obtained from spirometry for the healthy and asthmatic groups analyzed in the present study.

	Healthy Volunteers (n = 5)	Patients with Severe Asthma (n = 5)
Age at the date of entry [y] ^(1)^	22 (19–25)	43 (41–46)
Sex ^(2)^		
Male	40 (2)	40 (2)
Female	60 (3)	60 (3)
Age [y] ^(3)^	20.50 (19–22)	43.50 (43–45)
Male	22.30 (22–25)	43.33 (41–46)
Female		
Body Mass Index [Kg/m^2^] ^(1)^	24.3 (19.4–33.0)	33.4 (24.6–38.8)
Duration of Asthma [y] ^(3)^	NA	24.8 (8.0–45.0)
Immunoglobulin E [IU/mL] ^(1)^	34.8 (5.7–312.0)	187.0 (126.0–579.0) *
Percentage of peripheral blood eosinophils in blood ^(1)^	1.1 (0.5–5.5)	1.7 (0.6–2.1)
Percentage of blood eosinophils in sputum ^(1)^	0.0 (0.0–1.4)	3.5 (0.0–11.8)
Atopy, with positive allergy skin ^(2)^	80 (4)	100 (5)
FEV_1_ pre-BD use [L] ^(4)^	3.46 (2.36–5.12)	2.25 (1.83–2.45)
FEV_1_ post BD use [L] ^(4)^	4.87 (3.21–6.54)	3.70 (2.39–5.64)
FEV_1_ predicted ^(5)^	99.44 (83.90–102.81)	73.15 (56.48–91.41)

FEV_1_ = forced expiratory volume in the 1^st^ second of expiration.

NA = Not Applicable

BD = Bronchodilator

^(1)^ Data are medians and numbers in parentheses are minimum and maximum values.

^(2)^ Data are percentage and numbers in parentheses are numbers of subjects.

^(3)^ Data are means, with data ranges in parentheses.

^(4)^ Data are median with minimum and maximum values in parentheses.

^(5)^ Data are percentage and numbers in parentheses are minimum and maximum values.

* Immunoglobulin E level was reported in 3 of 5 patients with asthma

CT scans (Definition; Siemens, Munich, Germany) were performed with the following settings: 0.5–0.7 mm sections, 80–145 mAs according to body mass index, 0.4–0.7 mm in-plane resolution, and a 512x512 matrix.

The protocol of CT image acquisition received the approval of the institutional review board of Washington University (St Louis, MO). Informed consent was obtained from all subjects.

### Bronchial tree reconstruction

Tracheobronchial trees were segmented from CT images at FRC and TLC via a 3D confidence connected region growing procedure [18].

Airways geometry was obtained using the approach described in Miyawaki et al. [19] and was characterized by straight branches and circular cross sections. The length and the diameter of each branch were computed as in [19], i.e. the length of the branch was defined as the absolute distance between the centroids of the proximal and the distal cross section. The diameter of the branch was defined by the averaging the values of the diameters of the proximal and the distal cross sections. Analyses were performed down to the 4^th^ generation: generation 0 denoted the trachea, generation 1 the main bronchi, generation 2 the lobar bronchi, generation 3 and 4 the segmental and sub-segmental bronchi, respectively.

The cartilage and soft tissue fractions were assigned by matching the closest Horsfield order segment taking as a reference the values in [6]. The same procedure was applied to assign the thickness values for the control subjects. To account for the heterogeneity in thickness variation along different generations, the assignment of the thickness for the asthmatic subjects was accomplished using literature data from Montesantos et al. [20].

### Acoustic model

The model is based on the hypothesis of 1D wave propagation on asymmetrical, self-consistent branching networks (27), modified to allow the computation of wave propagation on a segment to segment basis to account for CT-based patient-specific geometries (11). The model aims at simulating an insonification experiment in an excised bronchial tree. In terms of mechanical characterization of the tracheobronchial tree, the material properties for the model are reported in Table 2.

**Table 2:** Material properties of the human airway tree model.

Variable	Units	Value
Air density (ρ_g_)	kg/m^3^	1.14
Air viscosity (η_g_)	Pa·s	1.86 × 10^−5^
Speed of sound in air (C_g_)	m/s	343.00
Airway Wall Elastic Modulus (E_w_)	Pa	5.81 × 10^4^ for soft tissue 3.92 × 10^6^ for cartilage
Airway Wall Viscosity (η_w_)	Pa·s	102.00 for soft tissue 688.00 for cartilage

The acoustic pressure planar wave could be introduced virtually everywhere in the bronchial tree. However, for the purpose of this work and to allow an easier comparison with [13] the acoustic wave was introduced as a non-homogeneous boundary condition at the inlet of the trachea, simulating an insonification experiment from an external source. The amplitude of the wave was chosen equal to 1 Pa and the frequencies analyzed were 200 Hz and 600 Hz as they can be considered relevant for respiratory sounds [21]: in particular, abnormal respiratory sounds such as crackles [22,23], wheezes [22], stridor and pleural rub [1] have their frequency content completely or partially included in the chosen range. Additionally, those frequencies belong to the range evaluated in the validation phase of the model [13].

The acoustic pressure at a point x was computed according to [13]:

$$P^{\left(n\right)}[x,\omega ]=\frac{{\;Z}_{0}^{\left(n\right)}[\omega ]}{\text{sinh}\;\left({\;\gamma }_{0}^{\left(n\right)}[\omega ]l\right)}(\frac{P_{in}^{\left(n\right)}[\omega ]}{{\;Z}_{in}^{\left(n\right)}[\omega ]}\text{cosh}\;\left({\;\gamma }_{0}^{\left(n\right)}[\omega ](x-l)\right)-\frac{P_{T}^{\left(n\right)}[\omega ]}{{\;Z}_{T}^{\left(n\right)}[\omega ]}\text{cosh}\;\left({\;\gamma }_{0}^{\left(n\right)}[\omega ]x\right))$$

where the terms ${\;Z}_{0}^{\left(n\right)}[\omega ]$ and ${\;\gamma }_{0}^{\left(n\right)}[\omega ]$ denote the characteristic impedance and propagation coefficient and *ω* the angular frequency for the n^th^ order segment; $P_{in}^{\left(n\right)}[\omega ]$ and $P_{T}^{\left(n\right)}[\omega ]$ represent the segment proximal and distal pressures, respectively; ${\;Z}_{in}^{\left(n\right)}[\omega ]$ and ${\;Z}_{T}^{\left(n\right)}[\omega ]$ are the n^th^ order segment inlet and outlet impedances; *l* is the length of the segment.

### Acoustic parameters computation

The following parameters were investigated:

1. Acoustic pressure distribution. For each segment of length *l*, it was chosen to solve the acoustic pressure for 9 equally-spaced interpolating points starting from the inlet (*x* = 0) to the outlet (*x* = *l*).
2. Input and terminal acoustic impedance, which measure the opposition of the system to acoustic wave propagation.
3. The wall acoustic radial velocity, which represents the velocity at which the airway wall vibrates in the direction normal to the airway walls because of the acoustic pressure propagating in it. It was obtained for the terminal point of each segment as the ratio between the acoustic pressure and the wall specific acoustic impedance (defined as the acoustic impedance over the cross-sectional area of the cylindrical segment).

Reference [13] provides more detailed information on the computation of each parameter and on the definition of the boundary conditions of the model. For each parameter, the median values calculated on all the segments belonging to that generation were reported in absolute value.

**Fig 1:**
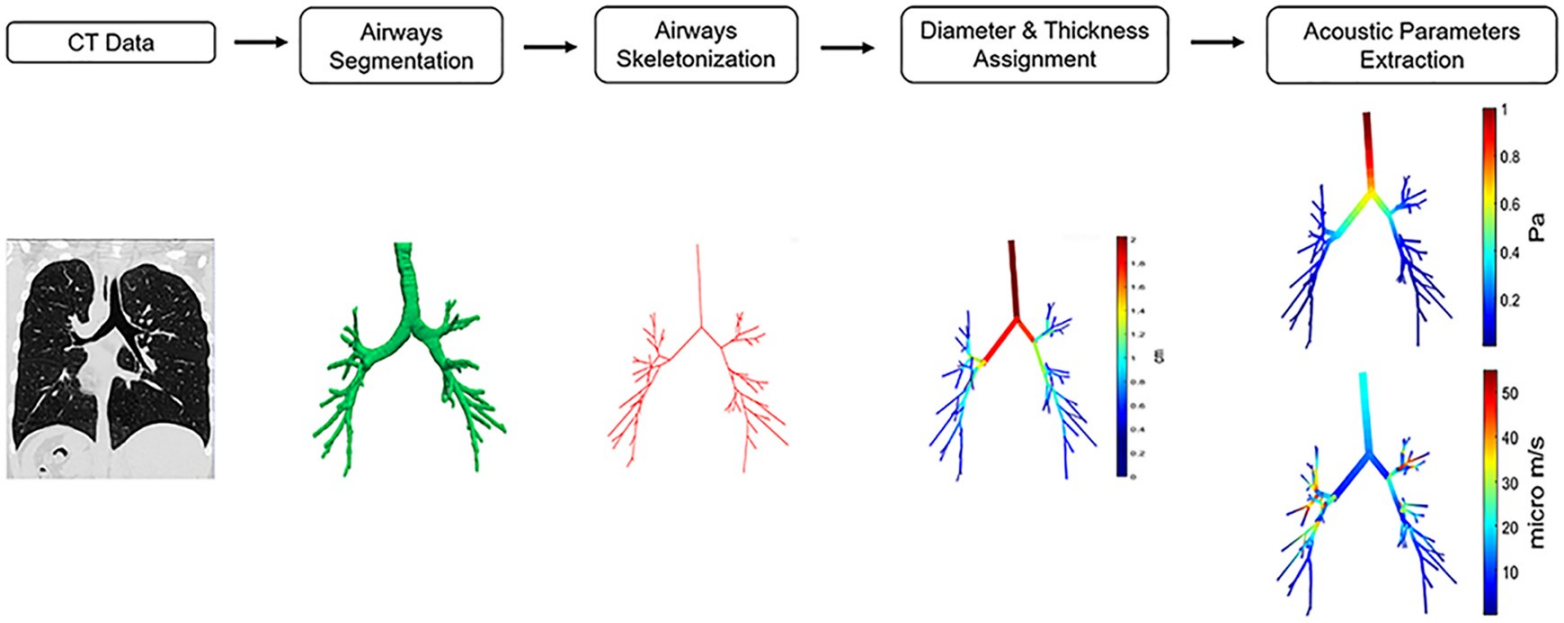
Algorithm for image processing and acoustic parameters estimation. 1) CT images are acquired at FRC and TLC; 2) airways are segmented via a 3D confidence connected region growing procedure; 3) The centerline of the airways is extracted and the diameters and length of each branch are computed according to [19]; 4) Thickness assignment is performed using literature data from [6] and [20], for healthy and asthmatic subjects, respectively; 5) The geometry is input to the model and the acoustic parameters are computed.

Fig 1 summarizes the steps of the image processing algorithm and the acoustic parameters’ estimation: 1) CT images acquired at FRC and TLC; 2) tracheobronchial tree segmentation via 3D confidence connected region growing procedure; 3) centerline extraction and branch measuring; 4) thickness assignment; 5) acoustic parameters computation. The geometry reconstruction and the model application were implemented using Matlab^®^ (r2018a, The MathWorks Inc., Natick, MA, 2018).

### Data analysis

One-way analysis of variance (ANOVA) was applied to separately compare different generations among healthy and asthmatic subjects. When the hypotheses of equal variance test and /or normality of the data distribution were rejected, nonparametric Kruskal–Wallis one-way ANOVA on ranks was applied.

To assess the differences between the two groups for each generation post-hoc tests were performed: Holm–Sidak’s and Dunn’s methods were used for parametric and nonparametric ANOVA tests, respectively. Significance was determined using a difference with P<0.05.

The alterations of the asthma group were also quantified in terms of percentual relative reduction or increment with respect to the control population by considering the median values of the two groups for each generation. Statistical analysis was performed in SigmaPlot (Systat Software, San Jose, CA).

## Results

### Analysis of the diameters

**Fig 2:**
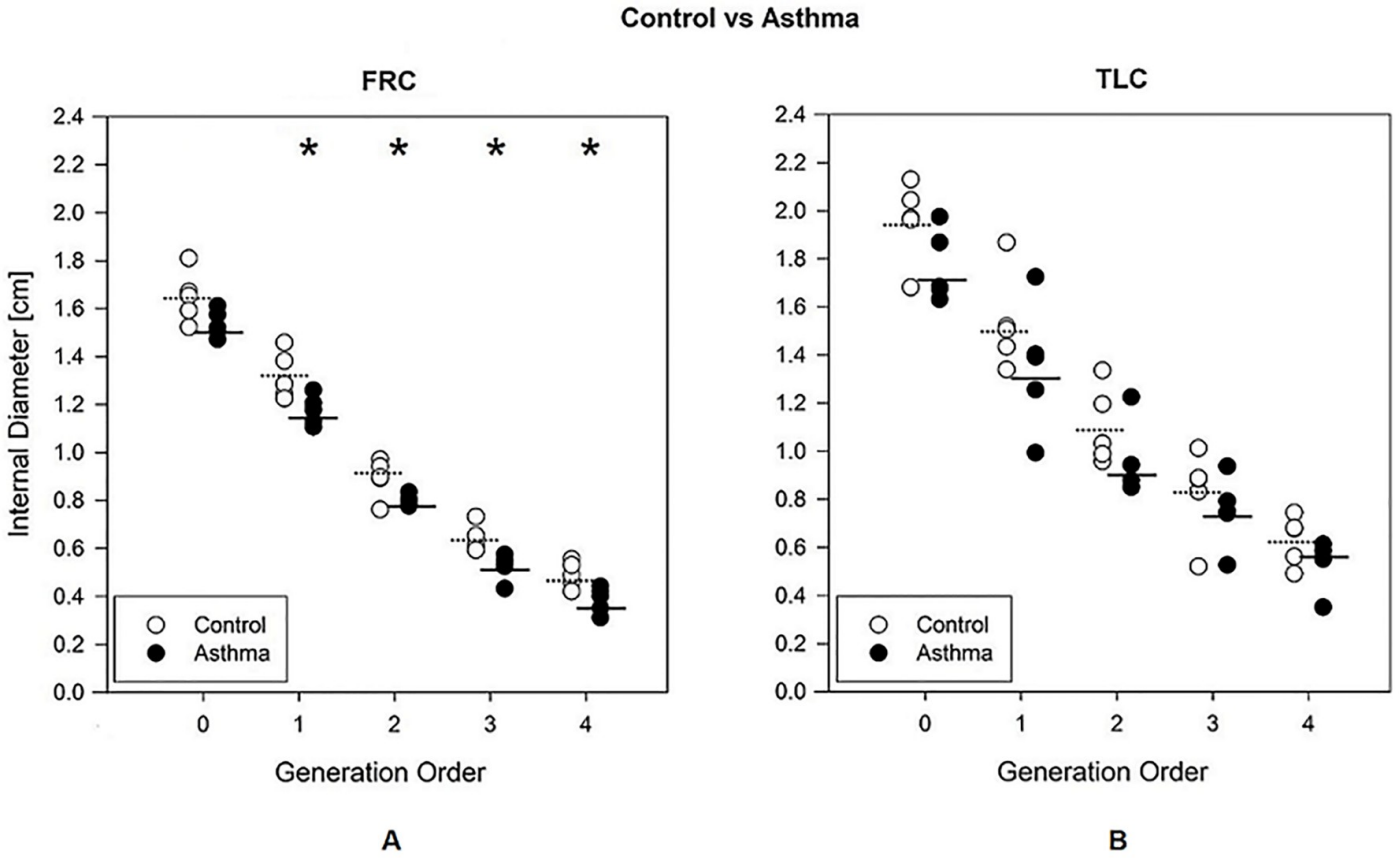
Internal diameter of the airways. Internal diameter of the airways as a function of the airway generation order for control (white circles) and asthma (black circles) groups, measured at FRC (A) and TLC (B). The mean values are reported with a dashed line for the control group and with a continuous line for the asthmatic group. *, p<0.05 between control and asthma groups.

Fig 2 reports the internal diameters at FRC (left) and TLC (right) in the control (white) and the asthma (black) groups. At FRC diameters were lower in the asthma subjects than in controls with a relative constriction of 8.3% for generation 1 (p = 0.029), 11.6% for generation 2 (p = 0.036), 12.5% for generation 3 (p = 0.014) and 17.7% for generation 4 (p = 0.014). At TLC, no significant differences were found between the controls and the asthmatic groups. Data are detailed in the S1 Table.

### Acoustic variables analysis at FRC

**Fig 3:**
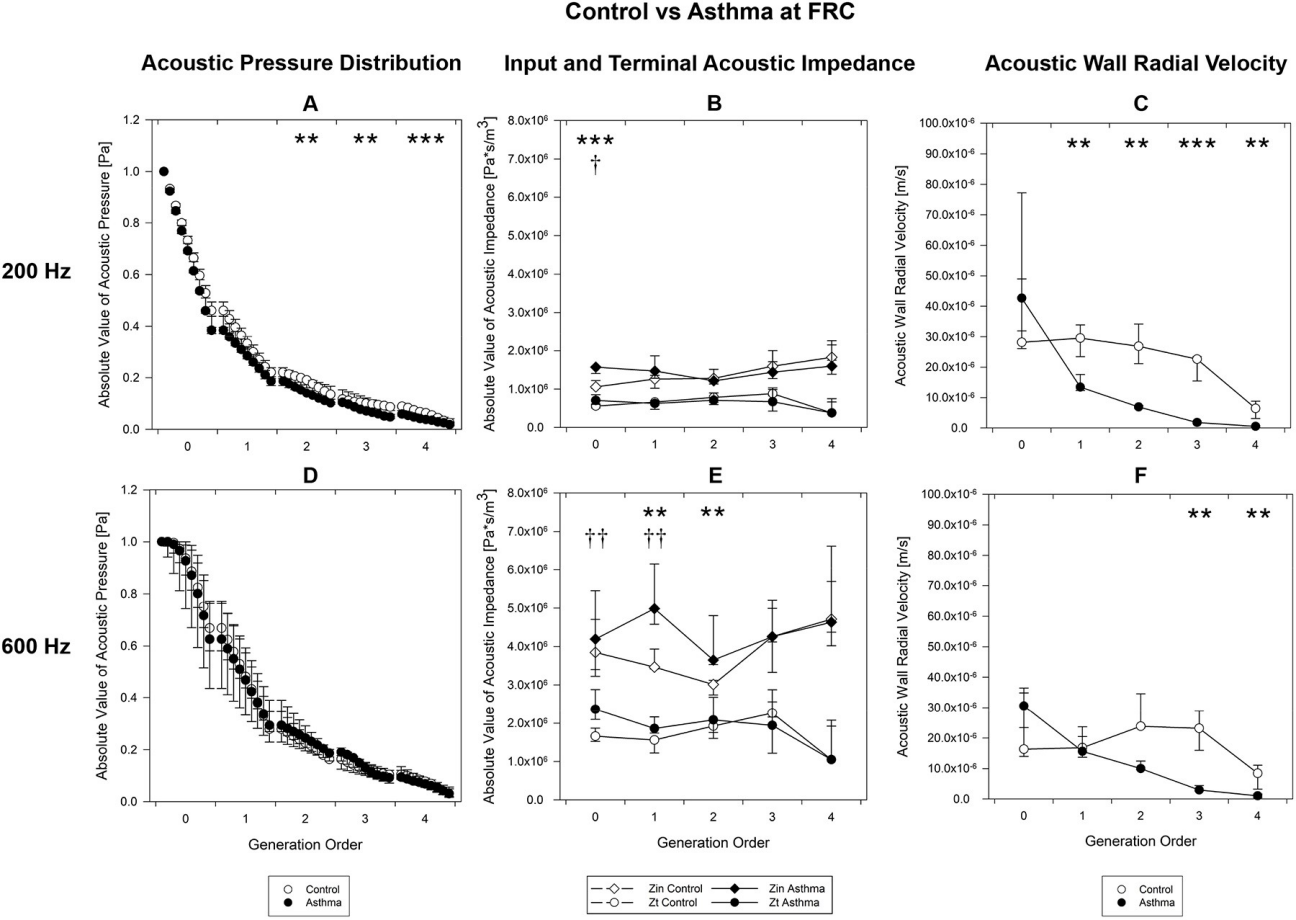
Acoustic parameters at FRC. Acoustic parameters computed at FRC, as a function of the airway generation order for control (white circles) and asthma (black circles) groups at 200 Hz (A, B, C) and 600 Hz (D, E, F). A, D) Absolute value of acoustic pressure distribution at 9 equally spaced points. B, E) Input and terminal impedances. C, F) Acoustic wall radial velocity at the distal end of the segments. The symbols indicate the median value for all the segments belonging to the given generation, the lower and the upper bars correspond to the 25^th^ percentile and the 75^th^ percentile, respectively. * or †, p<0.05; ** or ††, p<0.01, *** or †††, p<0.001 between health and asthma. for acoustic pressure, input (*) and terminal (†) impedances and wall radial velocity. FRC, Functional Residual Capacity.

Fig 3 shows the acoustic parameters obtained at FRC for the control (white) and the asthma (black) groups for each generation of the bronchial tree, both at 200 Hz (top) and 600 Hz (bottom).

In asthmatic patients, acoustic pressure was lower than in controls at 200 Hz (Fig 3A) for generations 2, 3 and 4 (relative reduction equal to 24.4% (p = 0.002), 25.4% (p = 0.003) and 39.3% (p<0.001), respectively). At 600 Hz (Fig 3D) no statistically relevant alterations induced by the pathology were identified and a high inter-variability in the asthmatic group was observed.

As regards the acoustic impedance, asthmatic population had higher input and terminal impedance than the control group. At 200 Hz (Fig 3B), significant differences were observed at the tracheal level for both the input and the terminal impedances with a relative increment of 48.6% (p<0.001) and 26.1% (p = 0.039), respectively. At 600 Hz (Fig 3E), significant differences were observed at the tracheal level for the terminal impedance (relative increment 42.4%, p = 0.006), at the generation 1 for both the input and terminal impedances (relative increment of 43.9%, p = 0.004 and 19.3%, p = 0.006, respectively), and at the generation 2 for the input impedance (relative increment 21.1%, p = 0.008).

In terms of acoustic wall radial velocity, the trend is similar between the frequencies under analysis: generation 0 shows the asthmatic group to have a higher velocity with respect to the healthy one, whereas from generation 1 an inversion of the trend is observed. The asthmatic group shows a lower wall radial velocity from generation 1 (relative reduction > = 54.5%, p< = 0.002) at 200 Hz (Fig 3C), and from generation 3 (relative reduction > = 87.2%, p = 0.008) at 600 Hz (Fig 3F). Data are detailed in the S2 Table.

### Acoustic variables analysis at TLC

**Fig 4:**
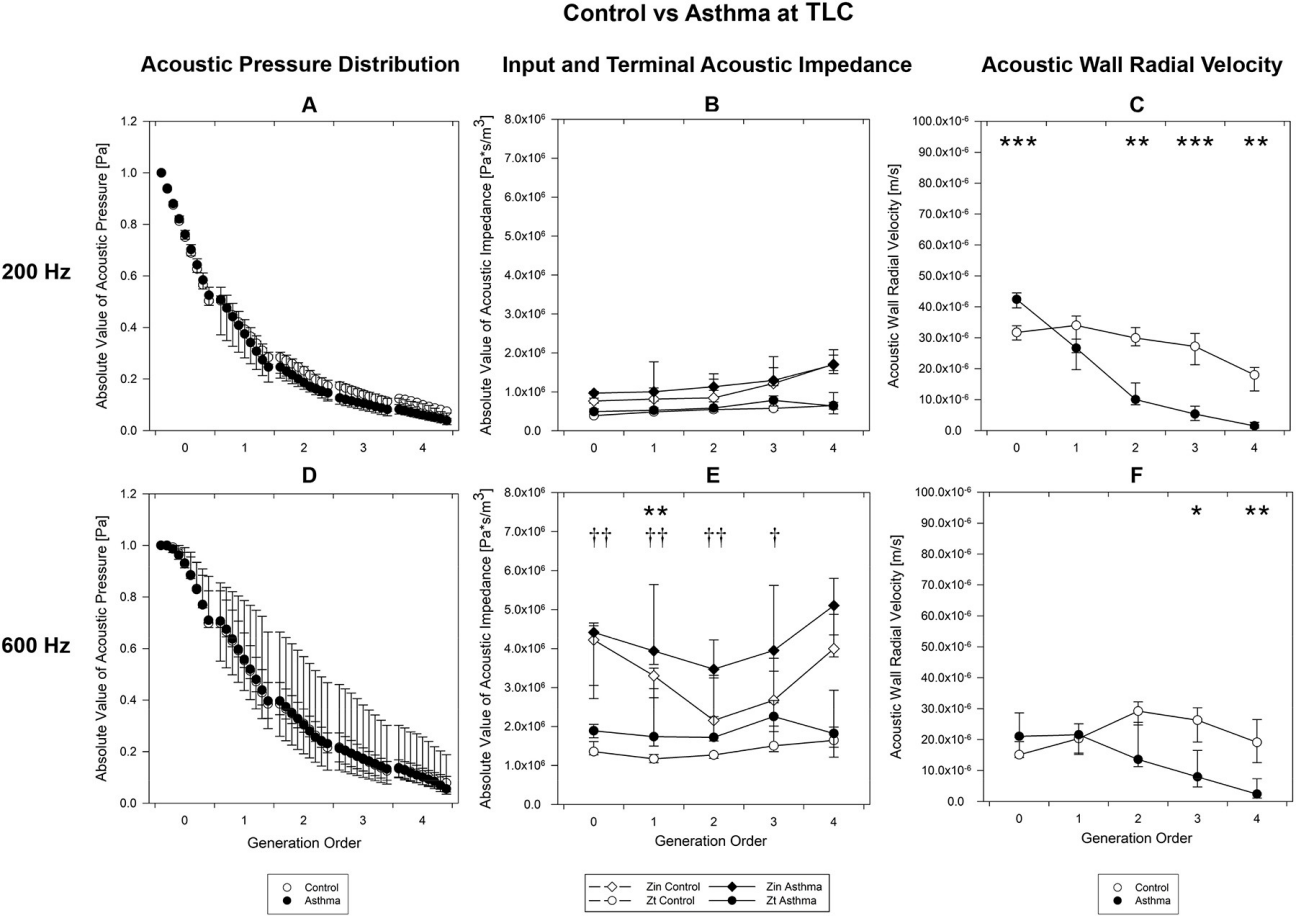
Acoustic parameters TLC. Acoustic parameters computed at TLC, as a function of the airway generation order for control (white circles) and asthma (black circles) groups at 200 Hz (A, B, C) and 600 Hz (D, E, F). A, D) Absolute value of acoustic pressure distribution at 9 equally spaced points. B, E) Input and terminal impedances. C, F) Acoustic wall radial velocity at the distal end of the segments. The symbols indicate the median value for all the segments belonging to the given generation, the lower and the upper bars correspond to the 25^th^ percentile and the 75^th^ percentile, respectively.* or †, p<0.05; ** or ††, p<0.01, *** or †††, p<0.001 between health and asthma. for acoustic pressure, input (*) and terminal (†) impedances and wall radial velocity. TLC, Total Lung Capacity.

Fig 4 shows the results obtained at TLC for the control (white) and asthma (black) groups at 200 Hz (top) and 600 Hz (bottom) as a function of the airway generation.

In terms of acoustic pressure (Fig 4A and 4D) no significant differences were found between the two groups. In general, the asthmatic group exhibited a higher variability with respect to the controls.

The acoustic impedance at 200 Hz (Fig 4B) showed no relevant differences. At 600 Hz (Fig 4E) asthmatic subjects showed higher acoustic impedance (both input and terminal) with respect to the control group. (Input impedance: relative increment of 19.3%, p = 0.008 for generation 1. Terminal Impedance: relative increment > = 35.7%, p<0.043 for the first 4 generations).

The acoustic wall radial velocity in the asthmatic group was higher in the trachea (p<0.001) and lower from generation 2 (relative reduction > = 66.5%, p< = 0.008) at 200 Hz (Fig 4C) while at 600 Hz (Fig 4F). velocity was lower from generation 3 (relative reduction > = 19.9%, p< = 0.018).

Data are detailed in the S3 Table.

## Discussion

In this work an analytical model of acoustic pressure propagation in the tracheobronchial tree was applied to CT data of a group of healthy and severe asthmatic subjects, both at FRC and TLC. The response of the system to an inlet acoustic pressure of 1 Pa of amplitude was evaluated at 200 Hz and 600 Hz, in terms of acoustic pressure distribution, acoustic impedance and acoustic wall radial velocity. Model was applied on a patient to patient basis and the results reported in terms of comparison between the asthmatic and healthy group to verify potential differences in the two cohorts. The results showed that the acoustic parameters were sensitive to the airways alterations induced by the asthma pathology, both at FRC and TLC.

Comparing the airways diameters between healthy and asthmatic subjects, significant differences were found between the two groups at FRC from the first generation, but not at TLC. These results confirmed previous evidences reported in literature: at TLC, the effect of the pathology is negligible due to the bronchodilatory effect of deep inspiration [24,25], whereas at FRC the increased collapsibility of the asthmatic airways [26,27] determines a lower diameter for the pathological subjects.

The acoustic pressure distribution was significantly lower in asthma at FRC at 200 Hz, but not at 600 Hz. This is related to the increased rigidity of the system with increasing frequency, causing the pressure to propagate more deeply in the tree so that an analysis limited to 5 generations might not suffice to highlight the discrepancy between the two populations. At TLC no significant variations between the two groups were recognized, probably due to the bronchodilation induced by inspiration in the asthmatics. However, the lack of significance in the results could possibly be ascribed also to the extremely high variability of the asthmatic group. Nonetheless, the fact that the inter-variability in the asthmatic group supports the evidence that asthma is a heterogenous disease and airways of the same order in different subjects can be affected with different modalities and severity [16,17].

The acoustic impedance was higher in the asthmatic subjects at both the respiratory volumes considered. This is particularly relevant for the terminal impedance and is in line to what is expected in [13]. In fact, the reduction of the diameter together with the thickening of the walls due to the pathology lead to a significant increment of the wall impedance, which is reflected in the acoustic impedance of the segment due to the direct proportionality between the two terms as reported in [13]. However, it is also verified that no specific trend can be identified as the statistical significance of the results is limited to first generations and, especially at FRC, it never extends to the successive ones, which theoretically are more likely to be affected by the pathology. Although the results of the acoustic impedance support the findings related to the pressure, they also show that, with the current approach, the use of acoustic impedance for the identification of specific features of the pathology requires further investigation.

The acoustic wall radial velocity was the parameter that better allowed the discrimination of the pathology and possible comparisons with previous findings. As a preliminary consideration, the results obtained are consistent to what is published in [13] for the frequencies of interest, taking in consideration that, in comparison to that work, the simulations have been run using a higher value for the elastic modulus of the cartilage component as observed in [28]. Acoustic wall radial velocity was shown to be significantly lower in the asthmatic case with respect to the healthy group for all the generations with the only exception of the trachea. The inverted trend at the level of the trachea can be possibly explained by considering that in the range between 380 and 560 Hz [11,29,30] resonance phenomena associated with the soft tissue component of trachea and main bronchi can occur, possibly affecting the results. The location of the resonance peak is dependent both on thickness and radius variations [12] and subject to a certain inter-variability. The increase in wall thickness and the reduction in diameter determined by the asthma pathology might have shifted the soft tissue resonance to lower frequencies thus explaining the observed inversion. However, further investigations are required to support this hypothesis. The fact that no difference at the tracheal level were found in [13] for the asthmatic case, could be possibly related to the simplified nature of the model applied for pathological simulation.

Asthma-induced heterogeneous bronchoconstriction creates a radius disparity between airway segments and generalized increment of acoustic impedances, which results in less acoustic energy reaching the higher generation of the bronchial tree as showed by the acoustic pressure distribution. This lower energy eventually causes the wall velocity to be lower for the pathological case. In terms of pathological features recognition, the choice of low frequency (200 Hz) and low respiratory volumes (FRC) seems to maximize the possibility of discriminating the asthmatic group with respect to the healthy one.

It is also worth noticing that the pressure drop was located mainly at the trachea and the main bronchi where approximately 70% of the pressure is dissipated. A similar trend can be observed for the acoustic wall radial velocity. It demonstrates that even if few generations are considered, these are enough to reflect the pathology-induced alterations along the tree for an insonification simulation. If this consideration is extended to those sounds generated inside the bronchial tree, which can be theoretically simulated with this model, the rapid pressure drop suggests that for those sounds originating from the central airways, a simplified geometry may suffice to simulate auscultative sounds. However, when adventitious lung sounds (for example crackles and wheezes) are considered, a more complex and complete system might be required as the sound generation generally occurs at the level of small airways [31]. Finally, asthma pathology is known to deeply affect small airways as confirmed also by the values in (Montesantos et al. 2013) used for the model, consequently the extension of the model to further branches can possibly result in even more relevant differences in the comparison with physiological cases.

In addition to the limitations of the acoustic model itself discussed in literature [13], some other geometry-specific issues can be recognized. The tracheobronchial trees were analyzed down to the 5^th^ generation, in line with most common automatic airways segmentation methods as reported in the literature [32,33]. Further software improvement to increase the sensitivity of the branch detection would provide a deeper insight in the tracheobronchial tree acoustics, thus allowing the identification of the pathology also in case of less severe conditions. Additionally, every segment was approximated with a cylindrical tube of constant radius and no physiological tempering nor natural curvature of the airways was considered, as the acoustic model requires a simplified geometry of the airways.

In the analysis of the asthmatic subjects, although the approach presented in this study accounted for the heterogeneity of the pathology in terms of radius and thickness variations, the simulations were affected by a lack of quantitative information regarding the alterations in terms of mechanical properties to which the airways undergo because of the onset and development of the pathology.

Finally, although both asthmatic and healthy populations include only adult subjects, the cohort of the study shows a mismatch between the age, body mass index and height of the two groups, which may contribute to confounding differences especially in airways sizes.

Future directions of developments include the necessity of collecting data regarding the mechanical properties of the constituent materials of the airway wall tissue especially in terms of their modification in response to the asthma pathology. Scanning Laser Doppler Vibrometry (SLDV) and *ex-vivo* mechanical characterization could be useful to this first task. Once collected, this information can be easily introduced in the model to enhance the precision of its predictions. Additionally, to provide a reliable comparison with the auscultation technique the model should be extended to account for the surrounding lung structure and the coupling with the chest surface to allow the simulation of the resulting sound field that reaches the surface of the chest [34]. Indeed, experimental studies have shown that there are multiple paths that sound energy can take in escaping from the airway lumen into the surrounding parenchymal tissue and chest wall to reach an acoustic sensor on the chest surface. Sound measurements on the torso surface over the lower lung lobes may in fact be dominated by sound energy that escaped the larger airways and traveled a longer path through surrounding tissue, thus complicating the analysis [35–36].

Finally, the coupling of this approach to the new emerging field of Magnetic Resonance Elastography (MRE), a noninvasive imaging technique to evaluate externally induced wave motion in the chest, would be of interest to better relate in-vivo geometrical, mechanical and acoustical properties of airways and lungs.

## Conclusions

In conclusion, this study provides an insight to the acoustic properties of the airways and to the alterations induced by the asthma condition, by simulating an insonification experiment at the level of the trachea. With respect to previous works, the application proposed in current approach accounts for the complexity and heterogeneity of asthmatic condition thanks to the use of patient-specific geometries and thickness data obtained from asthmatic subjects. The acoustic parameters analyzed showed a significant sensitivity to pathology induced alterations of the tracheobronchial tree, both at FRC and TLC. The application of the model to real pathological data demonstrates its reliability in a more realistic scenario than the ones previously analyzed in literature, highlighting, at the same time, the necessity of accounting for the heterogeneity of the alterations of the asthma pathology to properly model its complexity from both a geometrical and acoustical point of view.

The identification of possible diagnostic markers through this approach can be considered a preliminary attempt towards future clinical applications, where the study of acoustic wave propagation may provide specific acoustic features relevant either to quantify disease severity and treatment efficacy or to better characterize the mechanical properties of the in-vivo airways.

## Supporting information

S1 Table Internal diameter data. (DOCX)

S2 Table Acoustic parameters data at functional residual capacity (FRC). (DOCX)

S3 Table Computed acoustic data at total lung capacity (TLC). (DOCX)
