# Supplementary material for: Simulation of bronchial airway acoustics in healthy and asthmatic subjects
Source: PLoS One. 2020 Feb 10;15(2):e0228603. doi: 10.1371/journal.pone.0228603 (PMC7010248; doi:10.1371/journal.pone.0228603)
Supplement: S1 Table — (DOCX) [file pone.0228603.s001.docx]

**S1 – Internal** **Diameter Data**

Internal Diameter [cm] at Functional Residual Capacity (FRC)

|  | **Internal Diameter [cm]** | | | | | **Descriptive Statistics** | | | | |
| --- | --- | --- | --- | --- | --- | --- | --- | --- | --- | --- |
| **Healthy Subjects** | S1 | S2 | S3 | S4 | S5 | Mean | Median | Std Dev | Min | Max |
| Generation 0 | 1.670 | 1.653 | 1.592 | 1.522 | 1.810 | 1.649 | 1.653 | 0.107 | 1.522 | 1.810 |
| Generation 1 | 1.243 | 1.381 | 1.284 | 1.224 | 1.457 | 1.318 | 1.284 | 0.099 | 1.224 | 1.457 |
| Generation 2 | 0.763 | 0.971 | 0.942 | 0.892 | 0.897 | 0.893 | 0.897 | 0.080 | 0.763 | 0.971 |
| Generation 3 | 0.601 | 0.611 | 0.654 | 0.593 | 0.733 | 0.638 | 0.611 | 0.058 | 0.593 | 0.733 |
| Generation 4 | 0.556 | 0.460 | 0.488 | 0.421 | 0.531 | 0.491 | 0.488 | 0.054 | 0.421 | 0.556 |
| **Asthmatic Subjects** | S6 | S7 | S8 | S9 | S10 |  |  |  |  |  |
| Generation 0 | 1.611 | 1.575 | 1.472 | 1.507 | 1.520 | 1.537 | 1.520 | 0.056 | 1.472 | 1.611 |
| Generation 1 | 1.260 | 1.205 | 1.129 | 1.178 | 1.106 | 1.176 | 1.178 | 0.061 | 1.106 | 1.260 |
| Generation 2 | 0.806 | 0.781 | 0.776 | 0.835 | 0.793 | 0.798 | 0.793 | 0.023 | 0.776 | 0.835 |
| Generation 3 | 0.552 | 0.433 | 0.535 | 0.525 | 0.575 | 0.524 | 0.535 | 0.054 | 0.433 | 0.575 |
| Generation 4 | 0.442 | 0.402 | 0.422 | 0.352 | 0.312 | 0.386 | 0.402 | 0.053 | 0.312 | 0.442 |

Std Dev: Standard Deviation, Min: Minimum, Max: Maximum.

Diameter [cm] at Total Lung Capacity (TLC)

|  | **Internal Diameter [cm]** | | | | | **Descriptive Statistics** | | | | |
| --- | --- | --- | --- | --- | --- | --- | --- | --- | --- | --- |
| **Healthy Subjects** | S1 | S2 | S3 | S4 | S5 | Mean | Median | Std Dev | Min | Max |
| Generation 0 | 2.043 | 1.970 | 1.962 | 1.681 | 2.130 | 1.957 | 1.970 | 0.168 | 1.681 | 2.130 |
| Generation 1 | 1.433 | 1.519 | 1.506 | 1.339 | 1.868 | 1.533 | 1.506 | 0.200 | 1.339 | 1.868 |
| Generation 2 | 0.958 | 1.196 | 1.032 | 0.988 | 1.335 | 1.102 | 1.032 | 0.160 | 0.958 | 1.335 |
| Generation 3 | 0.832 | 0.884 | 0.888 | 0.521 | 1.013 | 0.827 | 0.884 | 0.184 | 0.521 | 1.013 |
| Generation 4 | 0.561 | 0.677 | 0.744 | 0.491 | 0.681 | 0.631 | 0.677 | 0.102 | 0.491 | 0.744 |
| **Asthmatic Subjects** | S6 | S7 | S8 | S9 | S10 |  |  |  |  |  |
| Generation 0 | 1.675 | 1.868 | 1.684 | 1.631 | 1.975 | 1.767 | 1.684 | 0.148 | 1.631 | 1.975 |
| Generation 1 | 1.403 | 0.993 | 1.392 | 1.255 | 1.725 | 1.354 | 1.392 | 0.265 | 0.993 | 1.725 |
| Generation 2 | 0.943 | 0.850 | 0.879 | 0.852 | 1.225 | 0.950 | 0.879 | 0.158 | 0.850 | 1.225 |
| Generation 3 | 0.792 | 0.527 | 0.747 | 0.742 | 0.938 | 0.749 | 0.747 | 0.147 | 0.527 | 0.938 |
| Generation 4 | 0.558 | 0.352 | 0.586 | 0.551 | 0.613 | 0.532 | 0.558 | 0.104 | 0.352 | 0.613 |

Std Dev: Standard Deviation, Min: Minimum, Max: Maximum.
