# Supplementary material for: Simulation of bronchial airway acoustics in healthy and asthmatic subjects
Source: PLoS One. 2020 Feb 10;15(2):e0228603. doi: 10.1371/journal.pone.0228603 (PMC7010248; doi:10.1371/journal.pone.0228603)
Supplement: S2 Table — (DOCX) [file pone.0228603.s002.docx]

**S2 – Acoustic parameters data at functional residual capacity (FRC)**

Acoustic Pressure [Pa] at frequency 200 Hz

| **Healthy Subjects** | **Interpolating Pt** | **G1** | **G2** | **G3** | **G4** | **G5** | **Asthmatic Subjects** | **Interpolating Pt** | **G1** | **G2** | **G3** | **G4** | **G5** |
| --- | --- | --- | --- | --- | --- | --- | --- | --- | --- | --- | --- | --- | --- |
| S1 | 1  2  3  4  5  6  7  8  9 | 1.000  0.941  0.881  0.821  0.761  0.701  0.641  0.582  0.522 | 0.522  0.488  0.454  0.419  0.385  0.350  0.316  0.281  0.246 | 0.246  0.232  0.218  0.203  0.189  0.175  0.161  0.146  0.132 | 0.116  0.111  0.106  0.103  0.100  0.097  0.094  0.092  0.089 | 0.089  0.085  0.081  0.076  0.067  0.060  0.052  0.043  0.035 | S6 | 1  2  3  4  5  6  7  8  9 | 1.000  0.926  0.853  0.780  0.707  0.634  0.561  0.488  0.415 | 0.415  0.388  0.362  0.335  0.308  0.280  0.253  0.226  0.198 | 0.198  0.185  0.172  0.159  0.146  0.133  0.119  0.106  0.094 | 0.094  0.087  0.082  0.076  0.071  0.065  0.060  0.050  0.044 | 0.053  0.049  0.045  0.042  0.038  0.035  0.032  0.030  0.027 |
| S2 | 1  2  3  4  5  6  7  8  9 | 1.000  0.932  0.864  0.796  0.728  0.660  0.593  0.526  0.459 | 0.459  0.426  0.393  0.361  0.328  0.296  0.264  0.232  0.201 | 0.201  0.192  0.184  0.176  0.167  0.159  0.151  0.142  0.134 | 0.134  0.128  0.122  0.116  0.110  0.104  0.098  0.092  0.086 | 0.086  0.080  0.074  0.067  0.063  0.055  0.044  0.034  0.025 | S7 | 1  2  3  4  5  6  7  8  9 | 1.000  0.922  0.845  0.767  0.690  0.612  0.535  0.457  0.380 | 0.380  0.356  0.332  0.308  0.284  0.259  0.235  0.210  0.186 | 0.186  0.174  0.163  0.151  0.139  0.131  0.121  0.110  0.100 | 0.106  0.097  0.087  0.077  0.067  0.060  0.054  0.047  0.039 | 0.049  0.046  0.042  0.038  0.035  0.032  0.029  0.025  0.022 |
| S3 | 1  2  3  4  5  6  7  8  9 | 1.000  0.917  0.837  0.759  0.684  0.611  0.540  0.470  0.401 | 0.401  0.380  0.358  0.336  0.315  0.293  0.271  0.249  0.228 | 0.228  0.220  0.213  0.206  0.198  0.191  0.184  0.176  0.169 | 0.169  0.160  0.150  0.141  0.132  0.122  0.113  0.103  0.093 | 0.094  0.087  0.077  0.068  0.058  0.049  0.039  0.029  0.018 | S8 | 1  2  3  4  5  6  7  8  9 | 1.000  0.917  0.836  0.757  0.677  0.599  0.520  0.441  0.362 | 0.362  0.338  0.314  0.290  0.265  0.241  0.216  0.191  0.166 | 0.166  0.158  0.150  0.143  0.135  0.127  0.120  0.112  0.104 | 0.104  0.097  0.090  0.082  0.075  0.068  0.065  0.061  0.058 | 0.060  0.054  0.047  0.041  0.035  0.030  0.024  0.018  0.013 |
| S4 | 1  2  3  4  5  6  7  8  9 | 1.000  0.908  0.821  0.738  0.658  0.580  0.505  0.432  0.360 | 0.360  0.340  0.321  0.302  0.282  0.263  0.243  0.224  0.204 | 0.204  0.198  0.191  0.184  0.178  0.171  0.165  0.158  0.152 | 0.107  0.102  0.097  0.092  0.086  0.081  0.076  0.070  0.065 | 0.084  0.078  0.069  0.061  0.052  0.044  0.035  0.026  0.016 | S9 | 1  2  3  4  5  6  7  8  9 | 1.000  0.932  0.864  0.796  0.729  0.662  0.595  0.527  0.460 | 0.460  0.428  0.395  0.362  0.329  0.296  0.262  0.228  0.194 | 0.194  0.181  0.167  0.153  0.140  0.126  0.116  0.109  0.101 | 0.101  0.093  0.084  0.075  0.067  0.059  0.055  0.052  0.048 | 0.059  0.055  0.051  0.047  0.042  0.035  0.029  0.020  0.015 |
| S5 | 1  2  3  4  5  6  7  8  9 | 1.000  0.932  0.865  0.797  0.729  0.661  0.593  0.526  0.458 | 0.458  0.426  0.394  0.362  0.330  0.298  0.267  0.235  0.204 | 0.204  0.191  0.178  0.165  0.152  0.138  0.125  0.112  0.099 | 0.102  0.099  0.097  0.093  0.090  0.087  0.083  0.080  0.077 | 0.077  0.072  0.068  0.066  0.064  0.062  0.055  0.050  0.047 | S10 | 1  2  3  4  5  6  7  8  9 | 1.000  0.905  0.816  0.732  0.650  0.570  0.491  0.412  0.333 | 0.333  0.310  0.288  0.265  0.242  0.219  0.196  0.173  0.149 | 0.149  0.142  0.135  0.128  0.121  0.114  0.106  0.099  0.092 | 0.092  0.087  0.082  0.076  0.071  0.066  0.061  0.056  0.051 | 0.075  0.069  0.061  0.052  0.044  0.036  0.027  0.022  0.016 |
| **Descriptive Statistics** | | | | | | | | | | | | | |
| Mean | | 0.715 | 0.328 | 0.177 | 0.103 | 0.059 |  | | 0.623 | 0.285 | 0.137 | 0.072 | 0.039 |
| Median | | 0.728 | 0.321 | 0.178 | 0.099 | 0.062 |  | | 0.689 | 0.284 | 0.135 | 0.071 | 0.038 |
| Std Deviation | | 0.186 | 0.008 | 0.031 | 0.023 | 0.020 |  | | 0.201 | 0.076 | 0.029 | 0.017 | 0.038 |
| Min | | 0.360 | 0.201 | 0.099 | 0.065 | 0.016 |  | | 0.333 | 0.149 | 0.092 | 0.039 | 0.013 |
| Max | | 1.000 | 0.522 | 0.246 | 0.169 | 0.094 |  | | 1.000 | 0.459 | 0.198 | 0.106 | 0.075 |

G: generation, Pt: Point, Std Dev: Standard Deviation, Min: Minimum, Max: Maximum.

Input and Terminal Acoustic Impedance [Pa*s/m^3^] at frequency 200 Hz

| **Healthy Subjects** | **Z** | **G0** | **G1** | **G2** | **G3** | **G4** | **Asthmatic Subjects** | **Z** | **G0** | **G1** | **G2** | **G3** | **G4** |
| --- | --- | --- | --- | --- | --- | --- | --- | --- | --- | --- | --- | --- | --- |
| S1 | Zin | 1.234e6 | 1.429e6 | 1.326e6 | 1.594e6 | 2.278e6 | S6 | Zin | 1.316e6 | 1.321e6 | 1.189e6 | 1.433e6 | 1.193e6 |
|  | Zt | 6.632e5 | 6.625e5 | 7.852e5 | 1.131e6 | 7.416e5 |  | Zt | 6.288e5 | 6.037e5 | 6.072e5 | 4.447e5 | 3.803e5 |
| S2 | Zin | 1.108e6 | 1.047e6 | 9.954e5 | 1.353e6 | 2.016e6 | S7 | Zin | 1.317e6 | 1.226e6 | 1.168e6 | 1.439e6 | 1.582e6 |
|  | Zt | 5.193e5 | 5.113e5 | 6.760e5 | 9.325e5 | 3.803e5 |  | Zt | 5.689e5 | 5.729e5 | 5.934e5 | 4.123e5 | 5.721e5 |
| S3 | Zin | 9.702e5 | 1.254e6 | 1.276e6 | 1.937e6 | 1.824e6 | S8 | Zin | 1.495e6 | 1.465e6 | 1.214e6 | 1.425e6 | 2.155e6 |
|  | Zt | 5.564e5 | 6.604e5 | 8.196e5 | 8.230e5 | 3.803e5 |  | Zt | 7.051e5 | 6.258e5 | 7.098e5 | 9.788e5 | 3.803e5 |
| S4 | Zin | 9.859e5 | 1.254e6 | 1.276e6 | 2.060e6 | 1.824e6 | S9 | Zin | 1.547e6 | 1.745e6 | 1.428e6 | 1.699e6 | 1.598e6 |
|  | Zt | 5.564e5 | 6.604e5 | 8.196e5 | 8.814e5 | 3.803e5 |  | Zt | 8.549e5 | 6.817e5 | 8.235e5 | 6.710e5 | 3.803e5 |
| S5 | Zin | 1.016e6 | 9.603e5 | 1.265e6 | 1.191e6 | 1.681e6 | S10 | Zin | 1.489e6 | 1.980e6 | 1.600e6 | 1.724e6 | 2.363e6 |
|  | Zt | 4.819e5 | 4.367e5 | 6.342e5 | 8.341e5 | 7.600e5 |  | Zt | 8.541e5 | 7.167e5 | 9.751e5 | 1.008e6 | 7.574e5 |
| **Descriptive Statistics** | | | | | | | | | | | | | |
| Mean | Zin | 1.063e6 | 1.189e6 | 1.228e6 | 1.627e6 | 1.925e6 |  | Zin | 1.433e6 | 1.547e6 | 1.320e6 | 1.544e6 | 1.778e6 |
|  | Zt | 5.554e5 | 5.862e5 | 7.469e5 | 9.205e5 | 5.285e5 |  | Zt | 7.224e5 | 6.402e5 | 7.418e5 | 7.030e5 | 4.941e5 |
| Median | Zin | 1.016e6 | 1.254e6 | 1.276e6 | 1.594e6 | 1.824e6 |  | Zin | 1.489e6 | 1.465e6 | 1.214e6 | 1.439e6 | 1.598e6 |
|  | Zt | 5.564e5 | 6.604e5 | 7.852e5 | 8.814e5 | 3.803e5 |  | Zt | 7.051e5 | 6.258e5 | 7.098e5 | 6.710e5 | 3.803e5 |
| Std Dev | Zin | 1.097e5 | 1.861e5 | 1.319e5 | 3.708e5 | 2.307e5 |  | Zin | 1.087e5 | 3.111e5 | 1.883e5 | 1.534e5 | 4.738e5 |
|  | Zt | 6.771e4 | 1.058e5 | 8.624e4 | 1.256e5 | 2.030e5 |  | Zt | 1.299e5 | 5.839e4 | 1.599e5 | 2.835e5 | 1.690e5 |
| Min | Zin | 9.702e5 | 9.603e5 | 9.954e5 | 1.191e6 | 1.681e6 |  | Zin | 1.316e6 | 1.226e6 | 1.168e6 | 1.425e6 | 1.193e6 |
|  | Zt | 4.819e5 | 4.367e5 | 6.342e5 | 8.230e5 | 3.803e5 |  | Zt | 5.689e5 | 5.729e5 | 5.934e5 | 4.123e5 | 3.803e5 |
| Max | Zin | 1.234e6 | 1.429e6 | 1.326e6 | 2.060e6 | 2.278e6 |  | Zin | 1.547e6 | 1.980e6 | 1.600e6 | 1.724e6 | 2.363e6 |
|  | Zt | 6.632e5 | 6.625e5 | 8.196e5 | 1.131e6 | 7.600e5 |  | Zt | 8.549e5 | 7.167e5 | 9.751e5 | 1.008e6 | 7.574e5 |

G: generation, Zin: Input Acoustic Impedance, Zt: Terminal Acoustic Impedance, Std Dev: Standard Deviation, Min: Minimum, Max: Maximum.

Acoustic Wall Radial Velocity [m/s] at frequency 200 Hz

| **Healthy Subjects** | **G0** | **G1** | **G2** | **G3** | **G4** | **Asthmatic Subjects** | **G0** | **G1** | **G2** | **G3** | **G4** |
| --- | --- | --- | --- | --- | --- | --- | --- | --- | --- | --- | --- |
| S1 | 3.550e-5 | 3.664e-5 | 4.149e-5 | 2.428e-5 | 8.527e-6 | S6 | 4.070e-5 | 2.077e-5 | 7.274e-6 | 2.234e-6 | 8.773e-7 |
| S2 | 3.131e-5 | 3.875e-5 | 2.542e-5 | 2.272e-5 | 6.453e-6 | S7 | 3.948e-5 | 1.851e-5 | 6.848e-6 | 1.311e-6 | 6.604e-7 |
| S3 | 8.802e-5 | 3.295e-5 | 2.865e-5 | 2.379e-5 | 3.202e-6 | S8 | 4.775e-5 | 1.415e-5 | 7.434e-6 | 1.785e-6 | 2.396e-7 |
| S4 | 7.891e-5 | 2.954e-5 | 2.569e-5 | 1.465e-5 | 2.871e-6 | S9 | 5.873e-5 | 1.505e-5 | 6.694e-6 | 1.859e-6 | 2.895e-7 |
| S5 | 3.484e-5 | 2.152e-5 | 1.964e-5 | 1.642e-5 | 9.575e-6 | S10 | 5.089e-5 | 1.319e-5 | 8.036e-6 | 2.414e-6 | 5.616e-7 |
| **Descriptive Statistics** | | | | | | | | | | | |
| Mean | 5.372e-5 | 3.188e-5 | 2.818e-5 | 2.037e-5 | 6.126e-6 |  | 4.751e-5 | 1.633e-5 | 7.257e-6 | 1.921e-6 | 5.257e-7 |
| Median | 3.550e-5 | 3.295e-5 | 2.569e-5 | 2.272e-5 | 6.453e-6 |  | 4.775e-5 | 1.505e-5 | 7.274e-6 | 1.859e-6 | 5.616e-7 |
| Std Dev | 2.739e-5 | 6.777e-6 | 8.125e-6 | 4.496e-6 | 3.038e-6 |  | 7.875e-6 | 3.192e-6 | 5.300e-7 | 4.288e-7 | 2.649e-7 |
| Min | 3.131e-5 | 2.152e-5 | 1.964e-5 | 1.465e-5 | 2.871e-6 |  | 3.948e-5 | 1.319e-5 | 6.694e-6 | 1.311e-6 | 2.396e-7 |
| Max | 8.802e-5 | 3.875e-5 | 4.149e-5 | 2.428e-5 | 9.575e-6 |  | 5.873e-5 | 2.077e-5 | 8.036e-6 | 2.414e-6 | 8.773e-7 |

G: generation, Std Dev: Standard Deviation, Min: Minimum, Max: Maximum.

Acoustic Pressure at frequency 600 Hz

| **Healthy Subjects** | **Interpolating Pt** | **G1** | **G2** | **G3** | **G4** | **G5** | **Asthmatic Subjects** | **Interpolating Pt** | **G1** | **G2** | **G3** | **G4** | **G5** |
| --- | --- | --- | --- | --- | --- | --- | --- | --- | --- | --- | --- | --- | --- |
| S1 | 1  2  3  4  5  6  7  8  9 | 1.000  0.995  0.980  0.953  0.915  0.867  0.808  0.741  0.666 | 0.666  0.622  0.576  0.530  0.483  0.435  0.386  0.337  0.288 | 0.288  0.268  0.248  0.229  0.210  0.192  0.173  0.156  0.138 | 0.127  0.121  0.115  0.109  0.105  0.101  0.098  0.094  0.090 | 0.090  0.086  0.082  0.078  0.069  0.061  0.053  0.043  0.034 | S6 | 1  2  3  4  5  6  7  8  9 | 1.000  1.000  1.000  1.000  0.968  0.920  0.856  0.776  0.682 | 0.682  0.646  0.608  0.568  0.526  0.482  0.437  0.391  0.343 | 0.343  0.321  0.298  0.276  0.253  0.231  0.208  0.185  0.167 | 0.167  0.152  0.142  0.132  0.123  0.113  0.103  0.086  0.076 | 0.091  0.085  0.078  0.071  0.065  0.060  0.056  0.051  0.047 |
| S2 | 1  2  3  4  5  6  7  8  9 | 1.000  0.985  0.959  0.922  0.873  0.815  0.747  0.670  0.587 | 0.587  0.544  0.500  0.456  0.413  0.368  0.324  0.280  0.237 | 0.237  0.225  0.214  0.202  0.191  0.180  0.170  0.159  0.148 | 0.148  0.141  0.133  0.126  0.119  0.111  0.104  0.097  0.090 | 0.090  0.084  0.077  0.071  0.065  0.059  0.048  0.036  0.026 | S7 | 1  2  3  4  5  6  7  8  9 | 1.000  0.998  0.981  0.950  0.904  0.842  0.766  0.676  0.575 | 0.575  0.543  0.510  0.475  0.440  0.403  0.366  0.329  0.290 | 0.290  0.272  0.255  0.237  0.222  0.205  0.188  0.171  0.154 | 0.169  0.155  0.139  0.123  0.107  0.094  0.083  0.073  0.061 | 0.076  0.072  0.066  0.061  0.056  0.051  0.045  0.039  0.034 |
| S3 | 1  2  3  4  5  6  7  8  9 | 1.000  0.925  0.850  0.775  0.700  0.625  0.550  0.474  0.399 | 0.399  0.376  0.352  0.329  0.305  0.281  0.256  0.232  0.208 | 0.208  0.200  0.192  0.184  0.176  0.169  0.161  0.153  0.145 | 0.145  0.136  0.127  0.118  0.109  0.100  0.091  0.083  0.074 | 0.078  0.070  0.063  0.058  0.050  0.041  0.033  0.024  0.015 | S8 | 1  2  3  4  5  6  7  8  9 | 1.000  0.961  0.916  0.863  0.802  0.733  0.656  0.571  0.481 | 0.481  0.452  0.423  0.393  0.361  0.329  0.296  0.262  0.228 | 0.228  0.218  0.208  0.197  0.187  0.176  0.166  0.155  0.142 | 0.142  0.132  0.122  0.114  0.104  0.094  0.090  0.085  0.080 | 0.083  0.074  0.066  0.057  0.049  0.041  0.033  0.025  0.018 |
| S4 | 1  2  3  4  5  6  7  8  9 | 1.000  0.920  0.841  0.761  0.682  0.602  0.522  0.443  0.363 | 0.363  0.342  0.321  0.299  0.277  0.255  0.233  0.211  0.189 | 0.189  0.182  0.175  0.168  0.160  0.153  0.146  0.139  0.132 | 0.086  0.081  0.077  0.072  0.067  0.063  0.058  0.054  0.049 | 0.071  0.064  0.057  0.052  0.045  0.037  0.030  0.022  0.014 | S9 | 1  2  3  4  5  6  7  8  9 | 1.000  0.963  0.922  0.876  0.825  0.769  0.707  0.642  0.572 | 0.572  0.537  0.501  0.463  0.424  0.383  0.341  0.298  0.254 | 0.254  0.236  0.219  0.202  0.184  0.166  0.155  0.145  0.135 | 0.135  0.123  0.112  0.100  0.089  0.078  0.073  0.068  0.063 | 0.076  0.071  0.065  0.060  0.055  0.046  0.037  0.026  0.018 |
| S5 | 1  2  3  4  5  6  7  8  9 | 1.000  1.000  1.000  0.984  0.950  0.904  0.844  0.772  0.690 | 0.690  0.646  0.601  0.554  0.506  0.458  0.408  0.357  0.306 | 0.306  0.286  0.265  0.243  0.222  0.201  0.179  0.158  0.137 | 0.146  0.142  0.137  0.133  0.128  0.124  0.120  0.115  0.111 | 0.111  0.103  0.095  0.088  0.083  0.079  0.072  0.063  0.060 | S10 | 1  2  3  4  5  6  7  8  9 | 1.000  0.968  0.930  0.885  0.832  0.769  0.697  0.616  0.527 | 0.527  0.495  0.462  0.427  0.393  0.357  0.322  0.285  0.249 | 0.249  0.238  0.228  0.217  0.206  0.195  0.183  0.171  0.159 | 0.159  0.151  0.142  0.133  0.124  0.115  0.106  0.097  0.088 | 0.131  0.117  0.102  0.088  0.074  0.060  0.046  0.036  0.026 |
| **Descriptive Statistics** | | | | | | | | | | | | | |
| Mean | | 0.802 | 0.395 | 0.192 | 0.110 | 0.061 |  | | 0.831 | 0.425 | 0.211 | 0.112 | 0.059 |
| Median | | 0.844 | 0.363 | 0.182 | 0.109 | 0.063 |  | | 0.863 | 0.423 | 0.206 | 0.112 | 0.059 |
| Std Deviation | | 0.185 | 0.137 | 0.044 | 0.027 | 0.024 |  | | 0.154 | 0.112 | 0.048 | 0.029 | 0.024 |
| Min | | 0.363 | 0.189 | 0.132 | 0.049 | 0.014 |  | | 0.480 | 0.228 | 0.135 | 0.061 | 0.018 |
| Max | | 1.000 | 0.690 | 0.307 | 0.148 | 0.111 |  | | 1.000 | 0.682 | 0.343 | 0.169 | 0.131 |

G: generation, Pt: Point, Std Dev: Standard Deviation, Min: Minimum, Max: Maximum.

Input and Terminal Acoustic Impedance [Pa*s/m^3^] at frequency 600 Hz

| **Healthy Subjects** | **Z** | **G0** | **G1** | **G2** | **G3** | **G4** | **Asthmatic Subjects** | **Z** | **G0** | **G1** | **G2** | **G3** | **G4** |
| --- | --- | --- | --- | --- | --- | --- | --- | --- | --- | --- | --- | --- | --- |
| S1 | Zin | 4.602e6 | 3.929e6 | 3.199e6 | 4.251e6 | 6.236e6 | S6 | Zin | 3.364e6 | 4.521e6 | 3.533e6 | 4.246e6 | 3.476e6 |
|  | Zt | 1.807e6 | 1.575e6 | 1.912e6 | 2.825e6 | 2.078e6 |  | Zt | 2.136e6 | 1.782e6 | 1.779e6 | 1.256e6 | 1.048e6 |
| S2 | Zin | 4.898e6 | 2.991e6 | 2.432e6 | 3.565e6 | 5.129e6 | S7 | Zin | 3.998e6 | 3.964e6 | 3.489e6 | 4.140e6 | 4.559e6 |
|  | Zt | 1.480e6 | 1.255e6 | 1.651e6 | 2.271e6 | 1.048e6 |  | Zt | 1.820e6 | 1.687e6 | 1.736e6 | 1.180e6 | 1.656e6 |
| S3 | Zin | 3.080e6 | 3.329e6 | 2.970e6 | 5.066e6 | 4.370e6 | S8 | Zin | 4.622e6 | 4.753e6 | 3.631e6 | 4.095e6 | 6.191e6 |
|  | Zt | 1.455e6 | 1.539e6 | 2.083e6 | 2.122e6 | 1.048e6 |  | Zt | 2.270e6 | 1.856e6 | 2.081e6 | 2.811e6 | 1.048e6 |
| S4 | Zin | 3.164e6 | 3.329e6 | 2.970e6 | 5.306e6 | 4.370e6 | S9 | Zin | 4.846e6 | 5.719e6 | 4.258e6 | 4.930e6 | 4.628e6 |
|  | Zt | 1.455e6 | 1.539e6 | 2.083e6 | 2.178e6 | 1.048e6 |  | Zt | 2.802e6 | 2.027e6 | 2.405e6 | 1.939e6 | 1.048e6 |
| S5 | Zin | 3.713e6 | 3.205e6 | 2.946e6 | 3.044e6 | 4.708e6 | S10 | Zin | 4.040e6 | 6.102e6 | 5.108e6 | 5.060e6 | 7.039e6 |
|  | Zt | 1.591e6 | 1.149e6 | 1.531e6 | 2.260e6 | 2.067e6 |  | Zt | 2.696e6 | 2.237e6 | 2.936e6 | 2.932e6 | 2.184e6 |
| **Descriptive Statistics** | | | | | | | | | | | | | |
| Mean | Zin | 3.891e6 | 3.357e6 | 2.903e6 | 4.246e6 | 4.962e6 |  | Zin | 4.174e6 | 5.012e6 | 4.004e6 | 4.494e6 | 5.179e6 |
|  | Zt | 1.558e6 | 1.411e6 | 1.852e6 | 2.331e6 | 1.458e6 |  | Zt | 2.345e6 | 1.918e6 | 2.187e6 | 2.024e6 | 1.397e6 |
| Median | Zin | 3.713e6 | 3.329e6 | 2.970e6 | 4.251e6 | 4.708e6 |  | Zin | 4.040e6 | 4.753e6 | 3.631e6 | 4.246e6 | 4.628e6 |
|  | Zt | 1.480e6 | 1.539e6 | 1.912e6 | 2.260e6 | 1.048e6 |  | Zt | 2.270e6 | 1.856e6 | 2.081e6 | 1.939e6 | 1.048e6 |
| Std Dev | Zin | 8.270e5 | 3.485e5 | 2.829e5 | 9.621e5 | 7.774e5 |  | Zin | 5.826e5 | 8.798e5 | 6.910e5 | 4.627e5 | 1.421e6 |
|  | Zt | 1.501e5 | 1.954e5 | 2.519e5 | 2.828e5 | 5.610e5 |  | Zt | 4.054e5 | 2.173e5 | 4.972e5 | 8.298e5 | 5.127e5 |
| Min | Zin | 3.080e6 | 2.991e6 | 2.432e6 | 3.044e6 | 4.370e6 |  | Zin | 4.846e6 | 6.102e6 | 5.108e6 | 5.060e6 | 7.039e6 |
|  | Zt | 1.455e6 | 1.149e6 | 1.531e6 | 2.122e6 | 1.048e6 |  | Zt | 2.802e6 | 2.237e6 | 2.936e6 | 2.932e6 | 2.184e6 |
| Max | Zin | 4.898e6 | 3.929e6 | 3.199e6 | 5.306e6 | 6.236e6 |  | Zin | 3.364e6 | 3.964e6 | 3.489e6 | 4.095e6 | 3.476e6 |
|  | Zt | 1.807e6 | 1.575e6 | 2.083e6 | 2.825e6 | 2.078e6 |  | Zt | 1.820e6 | 1.687e6 | 1.736e6 | 1.180e6 | 1.048e6 |

G: generation, Zin: Input Acoustic Impedance, Zt: Terminal Acoustic Impedance, Std Dev: Standard Deviation, Min: Minimum, Max: Maximum.

Acoustic Wall Radial Velocity [m/s] data at frequency 600 Hz

| **Healthy Subjects** | **G0** | **G1** | **G2** | **G3** | **G4** | **Asthmatic Subjects** | **G0** | **G1** | **G2** | **G3** | **G4** |
| --- | --- | --- | --- | --- | --- | --- | --- | --- | --- | --- | --- |
| S1 | 2.865e-5 | 2.513e-5 | 3.274e-5 | 2.293e-5 | 1.454e-5 | S6 | 3.484e-5 | 2.183e-5 | 1.122e-5 | 3.855e-6 | 1.588e-6 |
| S2 | 2.519e-5 | 2.902e-5 | 2.606e-5 | 2.470e-5 | 1.622e-5 | S7 | 3.157e-5 | 1.857e-5 | 9.989e-6 | 2.190e-6 | 1.120e-6 |
| S3 | 2.519e-5 | 2.902e-5 | 2.606e-5 | 2.470e-5 | 1.622e-5 | S8 | 3.426e-5 | 1.391e-5 | 9.257e-6 | 2.613e-6 | 3.544e-7 |
| S4 | 3.136e-5 | 2.247e-5 | 2.350e-5 | 1.144e-5 | 7.404e-6 | S9 | 4.030e-5 | 1.463e-5 | 8.677e-6 | 2.432e-6 | 3.950e-7 |
| S5 | 3.348e-5 | 1.773e-5 | 2.440e-5 | 3.024e-5 | 2.789e-5 | S10 | 3.773e-5 | 1.495e-5 | 9.832e-6 | 3.580e-6 | 8.846e-7 |
| **Descriptive Statistics** | | | | | | | | | | | |
| Mean | 2.877e-5 | 2.467e-5 | 2.655e-5 | 2.280e-5 | 1.646e-5 |  | 3.574e-5 | 1.678e-5 | 9.796e-6 | 2.934e-6 | 8.684e-7 |
| Median | 2.865e-5 | 2.513e-5 | 2.606e-5 | 2.470e-5 | 1.622e-5 |  | 3.484e-5 | 1.495e-5 | 9.832e-6 | 2.613e-6 | 8.846e-7 |
| Std Dev | 3.690e-6 | 4.771e-6 | 3.630e-6 | 6.922e-6 | 7.354e-6 |  | 3.361e-6 | 3.352e-6 | 9.506e-7 | 7.373e-7 | 5.171e-7 |
| Min | 2.519e-5 | 1.773e-5 | 2.350e-5 | 1.144e-5 | 7.404e-6 |  | 3.157e-5 | 1.391e-5 | 8.677e-6 | 2.190e-6 | 3.544e-7 |
| Max | 3.348e-5 | 2.902e-5 | 3.274e-5 | 3.024e-5 | 2.789e-5 |  | 4.030e-5 | 2.183e-5 | 1.122e-5 | 3.855e-6 | 1.588e-6 |

G: generation, Std Dev: Standard Deviation, Min: Minimum, Max: Maximum.
