# Supplementary material for: Simulation of bronchial airway acoustics in healthy and asthmatic subjects
Source: PLoS One. 2020 Feb 10;15(2):e0228603. doi: 10.1371/journal.pone.0228603 (PMC7010248; doi:10.1371/journal.pone.0228603)
Supplement: S3 Table — (DOCX) [file pone.0228603.s003.docx]

**S3 – Computed acoustic data at total lung capacity (TLC)**

Acoustic Pressure [Pa] at frequency 200 Hz

| **Healthy Subjects** | **Interpolating Pt** | **G1** | **G2** | **G3** | **G4** | **G5** | **Asthmatic Subjects** | **Interpolating Pt** | **G1** | **G2** | **G3** | **G4** | **G5** |
| --- | --- | --- | --- | --- | --- | --- | --- | --- | --- | --- | --- | --- | --- |
| S1 | 1  2  3  4  5  6  7  8  9 | 1.000  0.939  0.878  0.818  0.758  0.698  0.638  0.579  0.520 | 0.520  0.485  0.450  0.415  0.380  0.346  0.311  0.277  0.243 | 0.243  0.229  0.215  0.201  0.187  0.173  0.160  0.146  0.132 | 0.132  0.127  0.123  0.118  0.114  0.109  0.105  0.100  0.098 | 0.098  0.094  0.089  0.085  0.081  0.076  0.068  0.059  0.052 | S6 | 1  2  3  4  5  6  7  8  9 | 1.000  0.938  0.876  0.815  0.753  0.691  0.630  0.569  0.508 | 0.508  0.474  0.441  0.408  0.374  0.341  0.307  0.273  0.240 | 0.240  0.224  0.209  0.193  0.178  0.162  0.146  0.131  0.115 | 0.115  0.110  0.105  0.100  0.095  0.090  0.085  0.080  0.075 | 0.075  0.071  0.067  0.062  0.058  0.054  0.049  0.045  0.041 |
| S2 | 1  2  3  4  5  6  7  8  9 | 1.000  0.937  0.874  0.811  0.748  0.685  0.623  0.561  0.500 | 0.500  0.472  0.444  0.416  0.389  0.361  0.334  0.307  0.280 | 0.280  0.268  0.256  0.244  0.231  0.217  0.202  0.187  0.171 | 0.171  0.163  0.156  0.148  0.140  0.132  0.124  0.121  0.119 | 0.119  0.118  0.113  0.107  0.098  0.092  0.086  0.080  0.073 | S7 | 1  2  3  4  5  6  7  8  9 | 1.000  0.940  0.880  0.820  0.760  0.701  0.641  0.582  0.523 | 0.280  0.262  0.244  0.226  0.208  0.190  0.172  0.154  0.136 | 0.204  0.197  0.190  0.182  0.175  0.168  0.160  0.153  0.146 | 0.089  0.083  0.076  0.070  0.064  0.057  0.051  0.045  0.039 | 0.051  0.050  0.045  0.039  0.034  0.028  0.022  0.017  0.011 |
| S3 | 1  2  3  4  5  6  7  8  9 | 1.000  0.937  0.874  0.811  0.748  0.685  0.623  0.561  0.500 | 0.500  0.477  0.454  0.446  0.334  0.356  0.344  0.317  0.295 | 0.295  0.257  0.253  0.246  0.234  0.212  0.201  0.189  0.163 | 0.163  0.159  0.153  0.151  0.145  0.131  0.127  0.123  0.117 | 0.117  0.115  0.113  0.107  0.098  0.093  0.086  0.083  0.063 | S8 | 1  2  3  4  5  6  7  8  9 | 1.000  0.932  0.864  0.797  0.729  0.662  0.595  0.528  0.461 | 0.461  0.434  0.407  0.380  0.354  0.327  0.300  0.272  0.245 | 0.245  0.230  0.215  0.200  0.185  0.170  0.155  0.139  0.126 | 0.126  0.120  0.115  0.109  0.104  0.099  0.093  0.087  0.081 | 0.081  0.076  0.070  0.065  0.060  0.055  0.050  0.044  0.035 |
| S4 | 1  2  3  4  5  6  7  8  9 | 1.000  0.935  0.870  0.806  0.741  0.676  0.612  0.548  0.485 | 0.485  0.457  0.429  0.401  0.373  0.346  0.319  0.291  0.265 | 0.256  0.152  0.145  0.137  0.130  0.122  0.115  0.105  0.094 | 0.094  0.092  0.090  0.083  0.076  0.069  0.062  0.055  0.048 | 0.102  0.095  0.087  0.080  0.069  0.063  0.056  0.048  0.040 | S9 | 1  2  3  4  5  6  7  8  9 | 1.000  0.942  0.884  0.827  0.769  0.711  0.653  0.595  0.537 | 0.537  0.509  0.480  0.451  0.422  0.393  0.364  0.334  0.305 | 0.305  0.293  0.275  0.257  0.239  0.221  0.202  0.187  0.178 | 0.178  0.154  0.143  0.132  0.123  0.116  0.108  0.101  0.094 | 0.095  0.092  0.089  0.086  0.081  0.078  0.066  0.046  0.038 |
| S5 | 1  2  3  4  5  6  7  8  9 | 1.000  0.939  0.878  0.817  0.757  0.696  0.637  0.577  0.518 | 0.518  0.490  0.463  0.435  0.408  0.380  0.353  0.326  0.299 | 0.299  0.282  0.264  0.247  0.229  0.212  0.194  0.184  0.176 | 0.176  0.168  0.160  0.152  0.145  0.137  0.131  0.126  0.122 | 0.122  0.119  0.115  0.106  0.100  0.095  0.091  0.087  0.083 | S10 | 1  2  3  4  5  6  7  8  9 | 1.000  0.944  0.889  0.834  0.780  0.727  0.673  0.621  0.569 | 0.569  0.535  0.501  0.468  0.434  0.400  0.367  0.333  0.300 | 0.300  0.288  0.277  0.265  0.254  0.243  0.231  0.220  0.209 | 0.209  0.202  0.196  0.189  0.183  0.178  0.173  0.163  0.154 | 0.154  0.146  0.136  0.133  0.125  0.115  0.106  0.100  0.093 |
| **Descriptive Statistics** | | | | | | | | | | | | | |
| Mean | | 0.751 | 0.388 | 0.203 | 0.123 | 0.089 |  | | 0.758 | 0.358 | 0.206 | 0.114 | 0.069 |
| Median | | 0.748 | 0.380 | 0.202 | 0.126 | 0.091 |  | | 0.760 | 0.364 | 0.2020 | 0.105 | 0.065 |
| Std Dev | | 0.162 | 0.077 | 0.053 | 0.031 | 0.021 |  | | 0.158 | 0.111 | 0.049 | 0.044 | 0.034 |
| Min | | 0.485 | 0.243 | 0.094 | 0.048 | 0.040 |  | | 0.461 | 0.136 | 0.115 | 0.039 | 0.011 |
| Max | | 1.000 | 0.520 | 0.299 | 0.176 | 0.122 |  | | 1.000 | 0.569 | 0.305 | 0.209 | 0.154 |

G: generation, Pt: Point, Std Dev: Standard Deviation, Min: Minimum, Max: Maximum.

Input and Terminal Acoustic Impedance [Pa*s/m^3^] at frequency 200 Hz

| **Healthy Subjects** | **Z** | **G0** | **G1** | **G2** | **G3** | **G4** | **Asthmatic Subjects** | **Z** | **G0** | **G1** | **G2** | **G3** | **G4** |
| --- | --- | --- | --- | --- | --- | --- | --- | --- | --- | --- | --- | --- | --- |
| S1 | Zin | 7.573e5 | 9.883e5 | 9.815e5 | 1.246e6 | 1.703e6 | S6 | Zin | 9.305e5 | 1.003e6 | 1.022e6 | 1.025e6 | 1.267e6 |
|  | Zt | 4.149e5 | 4.710e5 | 5.461e5 | 8.618e5 | 6.705e5 |  | Zt | 4.942e5 | 4.691e5 | 5.449e5 | 6.173e5 | 6.902e5 |
| S2 | Zin | 7.372e5 | 7.939e5 | 8.380e5 | 1.196e6 | 1.548e6 | S7 | Zin | 9.673e5 | 2.325e6 | 1.338e6 | 2.187e6 | 1.696e6 |
|  | Zt | 3.816e5 | 4.832e5 | 5.546e5 | 5.765e5 | 6.463e5 |  | Zt | 5.402e5 | 1.393e6 | 5.500e5 | 6.557e5 | 3.803e5 |
| S3 | Zin | 7.372e5 | 7.939e5 | 8.380e5 | 1.196e6 | 1.548e6 | S8 | Zin | 9.773e5 | 9.968e5 | 1.132e6 | 1.296e6 | 1.677e6 |
|  | Zt | 3.816e5 | 4.832e5 | 5.546e5 | 5.765e5 | 6.463e5 |  | Zt | 4.788e5 | 5.278e5 | 5.846e5 | 8.212e5 | 4.877e5 |
| S4 | Zin | 1.088e6 | 1.165e6 | 1.932e6 | 1.988e6 | 2.373e6 | S9 | Zin | 1.044e6 | 1.222e6 | 1.319e6 | 1.624e6 | 1.803e6 |
|  | Zt | 5.441e5 | 6.896e5 | 4.358e5 | 4.639e5 | 4.671e5 |  | Zt | 5.853e5 | 6.557e5 | 7.706e5 | 7.830e5 | 6.371e5 |
| S5 | Zin | 6.373e5 | 6.931e5 | 7.722e5 | 1.138e6 | 1.794e6 | S10 | Zin | 7.489e5 | 9.625e5 | 1.052e6 | 1.288e6 | 2.088e6 |
|  | Zt | 3.437e5 | 4.000e5 | 5.388e5 | 7.631e5 | 7.889e5 |  | Zt | 4.705e5 | 5.248e5 | 7.231e5 | 9.802e5 | 1.277e6 |
| **Descriptive Statistics** | | | | | | | | | | | | | |
| Mean | Zin | 7.913e5 | 8.868e5 | 1.072e6 | 1.353e6 | 1.793e6 |  | Zin | 9.336e5 | 1.302e6 | 1.173e6 | 1.484e6 | 1.706e6 |
|  | Zt | 4.132e5 | 5.054e5 | 5.260e5 | 6.484e5 | 6.438e5 |  | Zt | 5.138e5 | 7.141e5 | 6.346e5 | 7.715e5 | 6.945e5 |
| Median | Zin | 7.372e5 | 7.939e5 | 8.380e5 | 1.196e6 | 1.703e6 |  | Zin | 9.673e5 | 1.003e6 | 1.132e6 | 1.296e6 | 1.696e6 |
|  | Zt | 3.816e5 | 4.832e5 | 5.461e5 | 5.765e5 | 6.463e5 |  | Zt | 4.942e5 | 5.278e5 | 5.846e5 | 7.830e5 | 6.371e5 |
| Std Dev | Zin | 1.722e5 | 1.887e5 | 4.868e5 | 3.573e5 | 3.408e5 |  | Zin | 1.111e5 | 5.811e5 | 1.480e5 | 4.465e5 | 2.953e5 |
|  | Zt | 7.743e4 | 1.087e5 | 5.084e4 | 1.606e5 | 1.151e5 |  | Zt | 4.820e4 | 3.857e5 | 1.049e5 | 1.444e5 | 3.480e5 |
| Min | Zin | 6.373e5 | 6.931e5 | 7.722e5 | 1.138e6 | 1.548e6 |  | Zin | 7.489e5 | 9.625e5 | 1.022e6 | 1.025e6 | 1.267e6 |
|  | Zt | 3.437e5 | 4.000e5 | 4.358e5 | 4.639e5 | 4.671e5 |  | Zt | 4.705e5 | 4.691e5 | 5.449e5 | 6.173e5 | 3.803e5 |
| Max | Zin | 1.088e6 | 1.165e6 | 1.932e6 | 1.988e6 | 2.373e6 |  | Zin | 1.044e6 | 2.325e6 | 1.338e6 | 2.187e6 | 2.088e6 |
|  | Zt | 5.441e5 | 6.896e5 | 5.546e5 | 8.618e5 | 7.889e5 |  | Zt | 5.853e5 | 1.393e6 | 7.706e5 | 9.802e5 | 1.277e6 |

G: generation, Zin: Input Acoustic Impedance, Zt: Terminal Acoustic Impedance, Std Dev: Standard Deviation, Min: Minimum, Max: Maximum.

Acoustic Wall Radial Velocity [m/s] at frequency 200 Hz

| **Healthy Subjects** | **G0** | **G1** | **G2** | **G3** | **G4** | **Asthmatic Subjects** | **G0** | **G1** | **G2** | **G3** | **G4** |
| --- | --- | --- | --- | --- | --- | --- | --- | --- | --- | --- | --- |
| S1 | 4.415e-5 | 3.823e-5 | 3.961e-5 | 2.911e-5 | 1.645e-5 | S6 | 5.247e-5 | 2.991e-5 | 8.194e-6 | 5.223e-6 | 1.543e-6 |
| S2 | 4.109e-5 | 4.606e-5 | 3.175e-5 | 3.486e-5 | 1.852e-5 | S7 | 5.450e-5 | 1.630e-5 | 1.051e-5 | 1.464e-6 | 1.613e-7 |
| S3 | 4.109e-5 | 4.606e-5 | 3.175e-5 | 3.486e-5 | 1.852e-5 | S8 | 4.802e-5 | 3.129e-5 | 1.012e-5 | 5.555e-6 | 1.683e-6 |
| S4 | 4.382e-5 | 3.774e-5 | 2.895e-5 | 1.583e-5 | 1.036e-5 | S9 | 5.363e-5 | 3.494e-5 | 1.084e-5 | 6.922e-6 | 1.485e-6 |
| S5 | 4.533e-5 | 2.353e-5 | 2.971e-5 | 2.857e-5 | 2.331e-5 | S10 | 5.959e-5 | 3.124e-5 | 2.259e-5 | 9.426e-6 | 3.674e-6 |
| **Descriptive Statistics** | | | | | | | | | | | |
| Mean | 4.310e-5 | 3.833e-5 | 3.235e-5 | 2.865e-5 | 1.743e-5 |  | 5.364e-5 | 2.874e-5 | 1.245e-5 | 5.718e-6 | 1.709e-6 |
| Median | 4.382e-5 | 3.823e-5 | 3.175e-5 | 2.911e-5 | 1.852e-5 |  | 5.363e-5 | 3.124e-5 | 1.051e-5 | 5.555e-6 | 1.543e-6 |
| Std Dev | 1.918e-6 | 9.204e-6 | 4.240e-6 | 7.770e-6 | 4.688e-6 |  | 4.156e-6 | 7.198e-6 | 5.761e-6 | 2.897e-6 | 1.259e-6 |
| Min | 4.109e-5 | 2.353e-5 | 2.895e-5 | 1.583e-5 | 1.036e-5 |  | 4.802e-5 | 1.630e-5 | 8.194e-6 | 1.464e-6 | 1.613e-7 |
| Max | 4.533e-5 | 4.606e-5 | 3.961e-5 | 3.486e-5 | 2.331e-5 |  | 5.959e-5 | 3.494e-5 | 2.259e-5 | 9.426e-6 | 3.674e-6 |

G: generation, Std Dev: Standard Deviation, Min: Minimum, Max: Maximum.

Acoustic Pressure [Pa] at frequency 600 Hz

| **Healthy Subjects** | **Interpolating Pt** | **G1** | **G2** | **G3** | **G4** | **G5** | **Asthmatic Subjects** | **Interpolating Pt** | **G1** | **G2** | **G3** | **G4** | **G5** |
| --- | --- | --- | --- | --- | --- | --- | --- | --- | --- | --- | --- | --- | --- |
| S1 | 1  2  3  4  5  6  7  8  9 | 1.000  0.993  0.976  0.949  0.912  0.866  0.810  0.746  0.674 | 0.674  0.630  0.584  0.537  0.489  0.440  0.390  0.340  0.290 | 0.290  0.269  0.249  0.229  0.209  0.190  0.171  0.152  0.134 | 0.134  0.129  0.123  0.117  0.112  0.107  0.101  0.096  0.091 | 0.091  0.087  0.083  0.079  0.075  0.072  0.068  0.058  0.045 | S6 | 1  2  3  4  5  6  7  8  9 | 1.000  0.975  0.945  0.908  0.866  0.819  0.767  0.710  0.649 | 0.649  0.615  0.578  0.540  0.500  0.459  0.416  0.372  0.327 | 0.327  0.306  0.286  0.265  0.244  0.223  0.202  0.180  0.159 | 0.159  0.152  0.145  0.138  0.131  0.124  0.118  0.111  0.104 | 0.104  0.098  0.092  0.086  0.080  0.074  0.068  0.062  0.056 |
| S2 | 1  2  3  4  5  6  7  8  9 | 1.000  0.980  0.953  0.933  0.848  0.814  0.756  0.638  0.625 | 0.625  0.531  0.523  0.514  0.466  0.442  0.412  0.334  0.312 | 0.312  0.302  0.294  0.282  0.258  0.246  0.234  0.224  0.182 | 0.182  0.176  0.167  0.155  0.148  0.143  0.136  0.133  0.112 | 0.112  0.105  0.100  0.095  0.090  0.082  0.074  0.071  0.063 | S7 | 1  2  3  4  5  6  7  8  9 | 1.000  0.981  0.956  0.924  0.887  0.844  0.796  0.743  0.684 | 0.369  0.349  0.328  0.306  0.284  0.261  0.238  0.214  0.189 | 0.284  0.274  0.264  0.254  0.244  0.234  0.224  0.214  0.203 | 0.125  0.117  0.108  0.099  0.090  0.081  0.072  0.063  0.054 | 0.075  0.072  0.064  0.056  0.048  0.040  0.032  0.024  0.015 |
| S3 | 1  2  3  4  5  6  7  8  9 | 1.000  0.980  0.951  0.913  0.868  0.814  0.754  0.688  0.615 | 0.615  0.581  0.546  0.510  0.474  0.437  0.400  0.362  0.325 | 0.325  0.308  0.291  0.275  0.258  0.241  0.224  0.204  0.185 | 0.185  0.174  0.164  0.154  0.145  0.135  0.126  0.114  0.102 | 0.112  0.100  0.097  0.095  0.090  0.082  0.073  0.068  0.063 | S8 | 1  2  3  4  5  6  7  8  9 | 1.000  0.978  0.949  0.912  0.867  0.814  0.755  0.689  0.618 | 0.618  0.589  0.558  0.526  0.492  0.458  0.422  0.386  0.348 | 0.348  0.328  0.308  0.287  0.266  0.245  0.223  0.202  0.185 | 0.185  0.177  0.168  0.160  0.151  0.144  0.136  0.128  0.120 | 0.120  0.111  0.102  0.095  0.088  0.081  0.073  0.062  0.048 |
| S4 | 1  2  3  4  5  6  7  8  9 | 1.000  0.986  0.961  0.925  0.878  0.822  0.756  0.682  0.601 | 0.601  0.563  0.524  0.484  0.444  0.403  0.362  0.321  0.280 | 0.280  0.166  0.137  0.127  0.118  0.109  0.100  0.092  0.083 | 0.083  0.077  0.070  0.064  0.058  0.052  0.046  0.040  0.034 | 0.075  0.067  0.060  0.054  0.046  0.043  0.038  0.035  0.028 | S9 | 1  2  3  4  5  6  7  8  9 | 1.000  0.976  0.946  0.911  0.870  0.824  0.773  0.716  0.656 | 0.656  0.625  0.593  0.561  0.527  0.493  0.457  0.421  0.385 | 0.385  0.367  0.345  0.323  0.300  0.277  0.254  0.237  0.226 | 0.213  0.200  0.186  0.172  0.159  0.149  0.140  0.131  0.121 | 0.121  0.118  0.115  0.112  0.106  0.101  0.082  0.058  0.048 |
| S5 | 1  2  3  4  5  6  7  8  9 | 1.000  0.996  0.984  0.965  0.938  0.903  0.861  0.813  0.758 | 0.758  0.732  0.701  0.667  0.630  0.590  0.546  0.501  0.453 | 0.453  0.426  0.398  0.370  0.342  0.314  0.295  0.278  0.262 | 0.262  0.247  0.233  0.219  0.205  0.191  0.178  0.167  0.158 | 0.158  0.153  0.148  0.143  0.129  0.121  0.115  0.109  0.102 | S10 | 1  2  3  4  5  6  7  8  9 | 1.000  1.000  1.000  1.000  1.000  0.998  0.985  0.967  0.945 | 0.945  0.928  0.904  0.874  0.837  0.793  0.743  0.688  0.628 | 0.628  0.610  0.591  0.572  0.552  0.532  0.510  0.488  0.466 | 0.466  0.454  0.441  0.429  0.411  0.390  0.366  0.344  0.322 | 0.322  0.312  0.299  0.290  0.272  0.251  0.238  0.230  0.215 |
| **Descriptive Statistics** | | | | | | | | | | | | | |
| Mean | | 0.864 | 0.497 | 0.242 | 0.134 | 0.086 |  | | 0.880 | 0.521 | 0.321 | 0.188 | 0.116 |
| Median | | 0.903 | 0.501 | 0.249 | 0.134 | 0.082 |  | | 0.912 | 0.500 | 0.277 | 0.145 | 0.088 |
| Std Deviation | | 0.123 | 0.123 | 0.088 | 0.055 | 0.031 |  | | 0.118 | 0.195 | 0.128 | 0.115 | 0.083 |
| Min | | 0.601 | 0.280 | 0.083 | 0.034 | 0.028 |  | | 0.618 | 0.189 | 0.159 | 0.054 | 0.015 |
| Max | | 1.000 | 0.758 | 0.453 | 0.262 | 0.158 |  | | 1.000 | 0.945 | 0.628 | 0.466 | 0.322 |

G: generation, Pt: Point, Std Dev: Standard Deviation, Min: Minimum, Max: Maximum.

Acoustic Impedance [Pa*s/m^3^] at frequency 600 Hz

| **Healthy Subjects** | **Z** | **G0** | **G1** | **G2** | **G3** | **G4** | **Asthmatic Subjects** | **Z** | **G0** | **G1** | **G2** | **G3** | **G4** |
| --- | --- | --- | --- | --- | --- | --- | --- | --- | --- | --- | --- | --- | --- |
| S1 | Zin | 3.781e6 | 2.942e6 | 2.238e6 | 2.936e6 | 4.539e6 | S6 | Zin | 4.415e6 | 3.612e6 | 3.141e6 | 3.022e6 | 3.709e6 |
|  | Zt | 1.227e6 | 1.081e6 | 1.210e6 | 2.093e6 | 1.809e6 |  | Zt | 1.757e6 | 1.410e6 | 1.623e6 | 1.814e6 | 2.007e6 |
| S2 | Zin | 3.856e6 | 2.323e6 | 2.085e6 | 2.595e6 | 3.715e6 | S7 | Zin | 4.277e6 | 7.014e6 | 4.495e6 | 6.391e6 | 5.103e6 |
|  | Zt | 1.117e6 | 1.139e6 | 1.250e6 | 1.496e6 | 1.632e6 |  | Zt | 1.944e6 | 3.971e6 | 1.634e6 | 1.924e6 | 1.048e6 |
| S3 | Zin | 3.856e6 | 2.323e6 | 2.085e6 | 2.595e6 | 3.715e6 | S8 | Zin | 4.500e6 | 3.574e6 | 3.472e6 | 3.824e6 | 4.997e6 |
|  | Zt | 1.117e6 | 1.139e6 | 1.250e6 | 1.496e6 | 1.632e6 |  | Zt | 1.664e6 | 1.589e6 | 1.723e6 | 2.384e6 | 1.381e6 |
| S4 | Zin | 4.881e6 | 3.166e6 | 4.313e6 | 4.511e6 | 5.186e6 | S9 | Zin | 4.668e6 | 3.940e6 | 3.950e6 | 4.845e6 | 5.226e6 |
|  | Zt | 1.483e6 | 1.372e6 | 1.121e6 | 1.201e6 | 1.291e6 |  | Zt | 1.891e6 | 1.973e6 | 2.279e6 | 2.257e6 | 1.822e6 |
| S5 | Zin | 2.843e6 | 2.908e6 | 2.067e6 | 2.493e6 | 3.922e6 | S10 | Zin | 1.835e6 | 4.275e6 | 3.353e6 | 3.953e6 | 6.375e6 |
|  | Zt | 1.370e6 | 9.978e5 | 1.240e6 | 1.917e6 | 2.162e6 |  | Zt | 2.174e6 | 1.741e6 | 2.225e6 | 2.962e6 | 3.858e6 |
| **Descriptive Statistics** | | | | | | | | | | | | | |
| Mean | Zin | 3.844e6 | 2.733e6 | 2.558e6 | 3.026e6 | 4.215e6 | 3.844e6 | Zin | 3.939e6 | 4.483e6 | 3.682e6 | 4.407e6 | 5.082e6 |
|  | Zt | 1.263e6 | 1.146e6 | 1.214e6 | 1.640e6 | 1.705e6 | 1.263e6 | Zt | 1.886e6 | 2.137e6 | 1.897e6 | 2.268e6 | 2.023e6 |
| Median | Zin | 3.856e6 | 2.908e6 | 2.085e6 | 2.595e6 | 3.922e6 | 3.856e6 | Zin | 4.415e6 | 3.940e6 | 3.472e6 | 3.953e6 | 5.103e6 |
|  | Zt | 1.227e6 | 1.139e6 | 1.240e6 | 1.496e6 | 1.632e6 | 1.227e6 | Zt | 1.891e6 | 1.741e6 | 1.723e6 | 2.257e6 | 1.822e6 |
| Std Dev | Zin | 7.216e5 | 3.865e5 | 9.837e5 | 8.468e5 | 6.392e5 | 7.216e5 | Zin | 1.185e6 | 1.443e6 | 5.428e5 | 1.284e6 | 9.469e5 |
|  | Zt | 1.612e5 | 1.389e5 | 5.480e4 | 3.590e5 | 3.166e5 | 1.612e5 | Zt | 1.951e5 | 1.046e6 | 3.271e5 | 4.525e5 | 1.092e6 |
| Min | Zin | 2.843e6 | 2.323e6 | 2.067e6 | 2.493e6 | 3.715e6 | 2.843e6 | Zin | 1.835e6 | 3.574e6 | 3.141e6 | 3.022e6 | 3.709e6 |
|  | Zt | 1.117e6 | 9.978e5 | 1.121e6 | 1.201e6 | 1.291e6 | 1.117e6 | Zt | 1.664e6 | 1.410e6 | 1.623e6 | 1.814e6 | 1.048e6 |
| Max | Zin | 4.881e6 | 3.166e6 | 4.313e6 | 4.511e6 | 5.186e6 | 4.881e6 | Zin | 4.668e6 | 7.014e6 | 4.495e6 | 6.391e6 | 6.375e6 |
|  | Zt | 1.483e6 | 1.372e6 | 1.250e6 | 2.093e6 | 2.162e6 | 1.483e6 | Zt | 2.174e6 | 3.971e6 | 2.279e6 | 2.962e6 | 3.858e6 |

G: generation, Zin: Input Acoustic Impedance, Zt: Terminal Acoustic Impedance, Std Dev: Standard Deviation, Min: Minimum, Max: Maximum.

Acoustic Wall Radial Velocity [m/s] at frequency 600 Hz

| **Healthy Subjects** | **G0** | **G1** | **G2** | **G3** | **G4** | **Asthmatic Subjects** | **G0** | **G1** | **G2** | **G3** | **G4** |
| --- | --- | --- | --- | --- | --- | --- | --- | --- | --- | --- | --- |
| S1 | 2.865e-5 | 2.513e-5 | 3.274e-5 | 2.293e-5 | 1.454e-5 | S6 | 3.429e-5 | 2.183e-5 | 9.286e-6 | 6.533e-6 | 2.209e-6 |
| S2 | 2.519e-5 | 2.902e-5 | 2.606e-5 | 2.470e-5 | 1.622e-5 | S7 | 3.634e-5 | 1.261e-5 | 1.309e-5 | 2.119e-6 | 2.356e-7 |
| S3 | 2.519e-5 | 2.902e-5 | 2.606e-5 | 2.470e-5 | 1.622e-5 | S8 | 3.280e-5 | 2.404e-5 | 1.249e-5 | 7.425e-6 | 2.373e-6 |
| S4 | 3.136e-5 | 2.247e-5 | 2.350e-5 | 1.144e-5 | 7.404e-6 | S9 | 3.387e-5 | 2.676e-5 | 1.255e-5 | 8.174e-6 | 1.913e-6 |
| S5 | 3.348e-5 | 1.773e-5 | 2.440e-5 | 3.024e-5 | 2.789e-5 | S10 | 4.929e-5 | 3.307e-5 | 3.160e-5 | 1.859e-5 | 8.395e-6 |
| **Descriptive Statistics** | | | | | | | | | | | |
| Mean | 2.877e-5 | 2.467e-5 | 2.655e-5 | 2.280e-5 | 1.646e-5 |  | 3.574e-5 | 1.678e-5 | 9.796e-6 | 2.934e-6 | 8.684e-7 |
| Median | 2.865e-5 | 2.513e-5 | 2.606e-5 | 2.470e-5 | 1.622e-5 |  | 3.484e-5 | 1.495e-5 | 9.832e-6 | 2.613e-6 | 8.846e-7 |
| Std Dev | 3.690e-6 | 4.771e-6 | 3.630e-6 | 6.922e-6 | 7.354e-6 |  | 3.361e-6 | 3.352e-6 | 9.506e-7 | 7.373e-7 | 5.171e-7 |
| Min | 2.519e-5 | 1.773e-5 | 2.350e-5 | 1.144e-5 | 7.404e-6 |  | 3.157e-5 | 1.391e-5 | 8.677e-6 | 2.190e-6 | 3.544e-7 |
| Max | 3.348e-5 | 2.902e-5 | 3.274e-5 | 3.024e-5 | 2.789e-5 |  | 4.030e-5 | 2.183e-5 | 1.122e-5 | 3.855e-6 | 1.588e-6 |

G: generation, Std Dev: Standard Deviation, Min: Minimum, Max: Maximum.
